# Anxiety symptoms and felt stigma among young people living with perinatally or behaviourally-acquired HIV in Ukraine: A cross-sectional survey

**DOI:** 10.1371/journal.pone.0210412

**Published:** 2019-01-24

**Authors:** Marion Durteste, Galyna Kyselyova, Alla Volokha, Ali Judd, Claire Thorne, Mario Cortina-Borja, Ruslan Malyuta, Violeta Martsynovska, Nataliya Nizova, Heather Bailey, the Study of Young People Living with HIV in Ukraine

**Affiliations:** 1 Population, Policy and Practice Programme, UCL Great Ormond Street Institute of Child Health, University College London, London, United Kingdom; 2 Shupyk National Medical Academy of Postgraduate Education, Kiev, Ukraine; 3 MRC Clinical Trials Unit at UCL, Institute of Clinical Trials & Methodology, University College London, London, United Kingdom; 4 Perinatal Prevention of AIDS Initiative, Odessa, Ukraine; 5 The Public Health Center of the Ministry of Health of Ukraine, Kiev, Ukraine; 6 Institute of Epidemiology and Infectious Diseases of NAMS, Kiev, Ukraine; Lluita contra la SIDA Foundation - Germans Trias i Pujol University Hospital - Autònoma de Barcelona University, SPAIN

## Abstract

**Background:**

Ukraine has the second largest European HIV epidemic. This study aimed to describe stigma, demographic and social factors and their association with anxiety among perinatally and behaviourally-HIV-infected (PHIV; BHIV) young people in Kiev and Odessa.

**Methods:**

104 PHIV and 100 BHIV young people aged 13–25 years completed a confidential tablet-based survey. Survey tools included the Hospital Anxiety and Depression Scale (HADS) (anxiety sub-scale scores of 8–10 indicating mild and ≥11 moderate/severe symptoms in last 7 days), Rosenberg Self-Esteem Scale (RSES) and HIV Stigma Scale (HSS) (short version, composite of disclosure, negative self-image and public attitudes sub-scales). Unadjusted Poisson regression models were fitted to explore factors associated with moderate/severe anxiety symptoms.

**Results:**

PHIV and BHIV young people were of median age 15.5 [IQR 13.9–17.1] and 23.0 [21.0–24.3] years, having registered for HIV care a median 12.3 [10.3–14.4] and 0.9 [0.2–2.4] years previously; 97% (97/100) and 66% (65/99) respectively were on ART. Overall 43% (95%CI 36–50%) reported any and 13% (95%CI 9–19%) moderate/severe anxiety symptoms, with no difference by HIV acquisition mode (*p* = 0.405) or gender (*p* = 0.700). 42% (75/180) reported history of an emotional health problem for which they had not been referred/attended for care. Moderate/severe anxiety symptoms were associated with HIV-related stigma (prevalence ratio (PR) 1.24 95%CI 1.14–1.34 per HSS unit increase), lower self-esteem (PR 0.83 95%CI 0.78–0.90 per RSES point increase), CD4 ≤350 cells/mm^3^ (PR 2.29 95%CI 1.06–4.97), having no-one at home who knew the respondent’s HIV status (PR 9.15 95%CI 3.40–24.66 vs all know) and, among BHIV, less stable living situation (PR 6.83 95%CI 1.99–23.48 for ≥2 vs no home moves in last 3 years) and history of drug use (PR 4.65 95%CI 1.83–11.85).

**Conclusions:**

Results indicated unmet need for psychosocial support. Further work is needed to explore strategies for mental health support, particularly around disclosure, self-esteem and stigma.

## Introduction

Ukraine has the second largest European HIV epidemic [1] with an estimated 238,000 people living with HIV (PLWH) overall and 5.2% of new infections in 2016 in the 15–24 age group [2]. As in other countries, an increasing number of perinatally HIV-infected (PHIV) children are reaching adolescence and young adulthood, with around 1500 PHIV young people aged 10–24 years registered for HIV care in 2016. These young people were born during a period when >50% of new HIV infections annually in Ukraine were in people who inject drugs (PWID) [2] and a substantial proportion therefore have exposure to parental substance use and/or AIDS-related illness or death within the family, and have spent time in non-parental care [3–5]. In 2016, there were also 11,560 behaviourally-HIV-infected (BHIV) young people aged 15–24 years nationally receiving HIV care (11,187 aged 20–24 years), three-quarters of whom had acquired HIV via sex and a quarter via injecting drug use (IDU) [6].

Adolescence and young adulthood are critical developmental periods marked by increasing importance of intimate relationships, social groups and body image, during which chronic illness can impact on puberty, psychological and cognitive functioning [7]. Findings from other settings have been inconsistent with regard to risk of mental health disorders among ageing cohorts of PHIV young people versus HIV-affected/exposed comparison groups [8–12]; some studies have found higher prevalence in both groups than the general population, highlighting the importance of contextual factors. Among BHIV youth, mental health problems may have contributed to vulnerability to HIV acquisition and/or be compounded by an HIV diagnosis, and there is some indication that this group is more vulnerable to depression and self-injurious and suicidal behaviours than PHIV youth [13, 14].

High levels of HIV-related stigma have been reported in Ukraine, including in medical settings, linked with initial concentration of the epidemic among PWID [15]. In the United States, Canada and Sweden, HIV-related stigma has been associated with poorer mental health, including depression/anxiety [16–18] and poorer quality of life [19, 20]. However, there are no published data on prevalence of mental health problems or felt stigma among young PLWH in Eastern Europe, and mental health disorders are poorly understood in the general population in Ukraine [21, 22]. The objectives of this study were to describe stigma, demographic and social factors and their association with anxiety among a group of young people with PHIV and BHIV receiving HIV care in two regions of Ukraine–Kiev and Odessa–which have a high burden of young people receiving HIV care [6].

## Methods

### Participants

From January 2016 to March 2017, young people aged 13–25 years attending Kiev and Odessa HIV/AIDS centres for routine HIV care self-completed an online survey via computer tablet using REDCap software [23]. Those eligible were: aware of their own HIV-positive status for at least six months and able to give informed consent (≥14 year olds) or assent, with consent from a parent/guardian (13 year olds, and 14–15 year olds unable to give informed consent themselves). Survey responses were submitted confidentially and anonymously linked to clinical data available in the Ukraine Paediatric HIV Cohort [3] or from medical note extraction using unique study numbers. Most likely mode of HIV acquisition was reported by the clinician; as only three young people with IDU-acquired infection completed the survey, their responses were excluded.

This study received ethics approval from the UCL Research Ethics Committee (3061/003) and Shupyk National Medical Academy of Postgraduate Education, Kiev, Ukraine, including for young people aged 14 or above to consent for themselves without involvement of a parent /guardian if competent to do so.

### Tools

Self-reported symptoms of anxiety and depression in the last 7 days were measured using the 14-item Hospital Anxiety and Depression Scale (HADS) [24, 25], with a score of 8–10 indicating mild and ≥11 moderate/severe symptoms on each of two sub-scales (anxiety and depression). In our study sample, the sub-scales had a Spearman’s rank correlation coefficient of 0.4441. Cronbach’s α was 0.7825 for the anxiety and 0.6486 for the depression sub-scale. Results on depression were therefore described as a point prevalence but not analysed further.

Young people were also asked: “*Have you or a loved one ever thought that you had low mood (depression)*, *fears and worries (anxiety)*, *aggression or anger*, *or any other emotional health issues*?” and about history of referral to or attendance at a service for an emotional health issue (questions from a UK study of young people with or affected by HIV [10]).

Self-esteem was measured using the Rosenberg Self-Esteem Scale (RSES) [26] (Cronbach’s α value 0.8005), with higher values indicating higher self-esteem. The Minneapolis-Manchester Quality of Life Instrument (MMQL) Adolescent Form [27] consists of 7 sub-scales, with higher scores indicating higher health-related quality of life (HRQOL). Cronbach’s α on each sub-scale was ≥0.7989 except for the intimate relations sub-scale (0.5526) which was excluded from analyses.

We used an abbreviated version of the HIV stigma scale [28], consisting of four areas: disclosure, negative self-image, public attitudes and personalised stigma [29], with higher scores indicating more severe stigma. Analyses of personalised stigma items were restricted to those young people who reported having ever disclosed their HIV status to someone. The total score across the other three areas gave a Cronbach’s α of 0.7814.

Hazardous drinking and/or alcohol use disorder was defined as a score of ≥3 in females and ≥4 in males on the Alcohol Use Disorders Identification Test (AUDIT-C) [30, 31]. The survey also included questions on socio-demographics, education and employment, disclosure of HIV status, smoking and substance use and use of support services. We used a Russian language translation of the HADS tool available from the publishers. For other tools and questions, two independent translations were undertaken and the translated versions of survey questions compared, to reach consensus about the most accurate wording. See S1 File for a copy of the questionnaire.

### Statistical analyses

Analyses comparing characteristics by mode of HIV acquisition and sex were conducted using the Chi-squared test or Fisher’s Exact test for categorical variables. Continuous scores on felt stigma, self-esteem and HRQOL were summarised as means to be consistent with the literature, with differences in location compared using the Wilcoxon-Mann-Whitney rank sum test due to non-normal distribution in some cases (assessed using the Shapiro-Wilk normality test).

Factors associated with moderate/severe anxiety were explored fitting univariable Poisson regression with robust standard errors due to the high prevalence of the outcome of moderate/severe anxiety [32]. Factors investigated in both PHIV and BHIV groups included age, death of parent(s), disclosure of young person’s HIV status to the people they were current living with, self-esteem, total stigma score across the disclosure, negative self-image and public attitudes sub-scales, and CD4 count. Poisson regression models were fitted restricted to the BHIV group only for number of home moves in the last 3 years, current smoking, hazardous drinking/alcohol use disorder, history of drug use, not being on ART and not being in education or employment, due to empty cells in the PHIV group and/or ≤5 PHIV young people reporting the exposure. Associations between anxiety/depression and efavirenz (EFV) use were explored due to the possible psychiatric side-effects of EFV. A minimally-adjusted model containing only stigma and self-esteem as explanatory variables was fitted, to explore the inter-correlation of these two factors and their association with moderate/severe anxiety symptoms.

Statistical analyses were conducted using SPSS version 22 and STATA SE version 15.0 (Stata Corp, College Station, Texas, USA).

## Results

The survey was completed by 104 PHIV young people (64 in Odessa, 40 in Kiev) and 100 young people with sexually-acquired HIV (“BHIV”, 56 in Odessa, 44 in Kiev). Of 41 BHIV males, 37 answered a question on their sexual partners, of whom 33 (89%) were men who have sex with men (MSM). Characteristics are shown in Table 1.

**Table 1:** Characteristics by mode of HIV acquisition.

	Total *n* = 204	PHIV *n* = 104	BHIV *n* = 100	*p*-value^1^
	n/N (%) or median [IQR]			
**Age**	19.0 [15.4;23.0]	15.5 [13.9;17.1]	23.0 [21.0;24.3]	<0.001
**Male**	98/204 (48)	57/104 (55)	41/100 (41)	0.048
**Death of biological parent(s)**	82/194 (42)	64/100 (64)	18/94 (19)	<0.001
**Living situation**^2^				
With parent(s)^3^	100/203 (49)	65/103 (63)	35/100 (35)	<0.001
With grandparent(s)/other adult family	56/203 (28)	47/103 (46)	9/100 (9)	<0.001
With spouse/partner	52/203 (26)	4/103 (4)	48/100 (48)	<0.001
With friend/housemate(s)	12/203 (6)	1/103 (1)	11/100 (11)	0.002
Alone	15/203 (7)	3/103 (3)	12/100 (12)	0.016
**Home moves in past 3 years**				
None	118/198 (60)	74/101 (73)	44/97 (45)	
1	59/198 (30)	20/101 (20)	39/97 (40)	
≥2	21/198 (11)	7/101 (7)	14/97 (14)	<0.001
**Disclosure within household**^4^				
All know young person’s HIV status	90/181 (50)	58/96 (60)	32/85 (38)	
Some know young person’s HIV status	76/181 (42)	31/96 (32)	45/85 (53)	
No one knows young person’s HIV status	15/181 (8)	7/96 (7)	8/85 (9)	0.008
**Ever disclosed their HIV status**	114/195 (58)	44/97 (45)	70/98 (71)	<0.001
**Education /employment**^2^				
Currently in education (full/part time)	113/199 (57)	91/99 (92)	22/100 (22)	<0.001
Currently in paid employment^5^	41/94 (44)	2/10 (20)	39/84 (46)	0.178
Not in education or employment	48/199 (24)	6/99 (2)	42/100 (42)	<0.001
**Use of support services**^2^				
Social services	112/204 (55)	49/104 (47)	63/100 (63)	0.023
Peer counselling	20/204 (10)	7/104 (7)	13/100 (13)	0.161
Adherence counselling	43/204 (21)	25/104 (24)	18/100 (18)	0.290
Support group	20/204 (10)	14/104 (13)	6/100 (6)	0.099
**Hazardous drinking/alcohol use disorder**	30/187 (16)	5/96 (5)	25/91 (27)	<0.001
**Cigarette smoking**				
Never smoked	86/191 (45)	63/97 (65)	23/94 (24)	
Smoked in the past	46/191 (24)	23/97 (24)	23/94 (24)	
Currently smoking	59/191 (31)	11/97 (11)	48/94 (51)	<0.001
**Ever used drugs recreationally**	19/191 (10)	4/97 (4)	15/94 (16)	0.007
**Time since HIV test (i.e. registration for HIV care at the centre)** (years)^6^	5.4 [0.9, 12.4]	12.3 [10.3, 14.4]	0.90 [0.2, 2.4]	<0.001
**Ever diagnosed with AIDS**	50/203 (25)	44/104 (42)	6/99 (6)	<0.001
**CD4 count** (cells/mm^3^)^7^	624 [416, 874]	785 [585, 997]	467 [314, 639]	<0.001
**CD4 count ≤350 cells/mm**^**3**^^7^	34/190 (18)	8/96 (8)	26/94 (28)	0.001
**On ART at time of survey completion**	162/199 (81)	97/100 (97)	65/99 (66)	<0.001

^**1**^. for comparison of proportions or medians between PHIV and BHIV groups

^**2**^. multiple responses could be selected or variables not mutually exclusive;

^**3**^. including adopted/foster/step parents;

^**4**^. excluding those living alone (*n* = 15), household defined as people the young person is currently living with

^**5**^. of those not in full time education;

^**6**^. All young people had first tested HIV-positive at least 6 months prior to completing the survey, but some had initiated HIV care more recently.

^**7**^ closest to survey completion (up to 7 months before or 30 days after survey completion)

Living circumstances partly reflected the age difference between PHIV and BHIV young people (median 15.5 years and 23.0 years respectively). The 42/100 BHIV young people not in education or employment included those looking for work (*n* = 14) or looking after home/family (*n* = 20). Of 51 BHIV females with data available, 20 were pregnant at the time of survey completion as were 2 of the PHIV group. Four PHIV young people (two female) and 32 BHIV (all female) were parents at the time of survey completion; 3/4 and 28/32 respectively were living with their child(ren). Smoking, history of illicit drug use and current hazardous drinking/alcohol use disorder were all more common among the BHIV young people (Table 1).

Nearly all the PHIV group were on ART, compared with around two-thirds of the BHIV group; median time since ART initiation was 8.7 [IQR 6.0, 11.5] years for the PHIV group (available for 96/97, equating to an age of 6.3 [IQR3.8, 10.3] years) and 0.6 [0.2, 1.8] years (available for 62 of the 65 on ART) for the BHIV. For those BHIV young people not yet on ART, median time since initiation of HIV care was 0.33 (IQR 0.11, 1.34) years vs 1.13 years (IQR 0.37, 2.84) for those on ART (*p* = 0.008). Of the 26 in the BHIV group with a CD4 count ≤350 cells/mm^3^, 9 had a CD4 count <200 cells/mm^3^ and 14 had started HIV care in the year preceding the survey. Of those on ART and with drug data available, 42% (41/97) of the PHIV group and 28% (18/65) of the BHIV group were on EFV-containing regimens.

### Measures of mental health, stigma, self-esteem and HRQOL

Table 2 shows symptoms of anxiety and depression in the last seven days and measures of HIV-related stigma, self-esteem and HRQOL by mode of HIV acquisition. Overall, 43% (95% CI 36–50%) reported any anxiety symptoms, and 13% (95% CI 9–19%) moderate/severe symptoms with no difference by mode of HIV acquisition. The proportion with any depression symptoms was 13% (95%CI 8–18%); of the 5% (95% CI 3–10%) with moderate/severe symptoms of depression, all 10 also had symptoms of anxiety (moderate/severe in 6/10).

**Table 2:** Mental health measures, stigma, self-esteem and health-related quality of life by mode of HIV acquisition.

	Total (*n* = 204)	PHIV (*n* = 104)	BHIV (*n* = 100)	*p*-value^e^
	*n/N* (%) or mean (SD)			
**Anxiety symptoms in last 7 days**^a^				
Mild anxiety (score 8–10)	56/188 (30)	27/95 (28)	29/93 (31)	
Moderate/severe anxiety (score ≥11)	25/188 (13)	10/95 (11)	15/93 (16)	0.405
**Depression symptoms in last 7 days**^a^				
Mild depression	14/188 (7)	11/94 (12)	3/94 (3)	
Moderate/severe depression	10/188 (5)	3/94 (3)	7/94 (7)	0.044
**HIV Stigma Scale**^b^				
Disclosure score (*n* = 186)	5.78 (1.54)	5.26 (1.63)	6.28 (1.28)	<0.001
Negative self-image score (*n* = 183)	6.78 (2.06)	6.24 (1.87)	7.29 (2.10)	0.001
Public attitudes score (*n* = 189)	5.89 (1.43)	5.53 (1.47)	6.25 (1.29)	0.001
Total score across disclosure, negative self-image and public attitudes sub-scales (*n* = 176)	18.48 (3.98)	17.00 (3.76)	19.80 (3.72)	<0.001
Personalised stigma score (*n* = 79)	6.77 (2.17)	6.16 (2.36)	7.06 (2.03)	0.048
**Self-esteem**^c^				
Total score	20.38 (4.07)	20.59 (4.12)	20.18 (4.02)	0.449
**Health-related quality of life**^d^				
Physical functioning score (*n* = 189)	3.50 (0.64)	3.60 (0.67)	3.40 (0.59)	0.024
Psychological functioning score (*n* = 189)	3.28 (0.57)	3.46 (0.55)	3.11 (0.54)	<0.001
Body image score (*n* = 193)	3.62 (0.73)	3.63 (0.74)	3.60 (0.73)	0.802
Social functioning score (*n* = 190)	3.72 (0.69)	3.76 (0.74)	3.67 (0.63)	0.325
Cognitive functioning score (*n* = 179)	3.61 (0.72)	3.48 (0.76)	3.75 (0.66)	0.013
Outlook on life score (*n* = 195)	3.66 (0.86)	3.75 (0.84)	3.56 (0.88)	0.224

^a^. measured using the Hospital Anxiety and Depression Scale (HADS) [24];

^b^. abbreviated version [29], higher scores indicate more severe stigma;

^c^. Rosenberg Self-Esteem Scale [26], higher scores indicate higher self-esteem

^d^. Minneapolis-Manchester Quality of Life Instrument Adolescent Form [27], higher scores indicate better health-related quality of life;

^e^. for comparison of proportions or differences in location between PHIV and BHIV groups

BHIV young people reported higher levels of HIV-related stigma than PHIV young people across all sub-scales and poorer physical and psychological functioning, but there was no difference between BHIV and PHIV groups in self-esteem or in HRQOL relating to body image, social functioning or outlook on life. BHIV reported better cognitive functioning than the PHIV group (Table 2). There was no difference in anxiety symptoms by gender (33% (29/89) of males and 27% (27/99) of females reported mild and 13% (12/89) and 13% (13/99) moderate/severe symptoms, *p* = 0.700). Stigma scores were also similar by gender across disclosure (*p* = 0.578), negative self-image (*p* = 0.458) and personalised stigma (*p* = 0.137) sub-scales, but females reported higher levels of stigma in the public attitudes domain (mean 6.20 (SD 1.43) vs 5.55 (1.35), *p* = 0.001). There was no difference between males and females in self-esteem scores (mean 20.6 (SD 4.04) for males vs 20.2 (SD 4.10) for females, *p* = 0.552) or in any measure of HRQOL (data not shown).

### Symptoms of anxiety in last 7 days and history of emotional health issues

**Fig 1:**
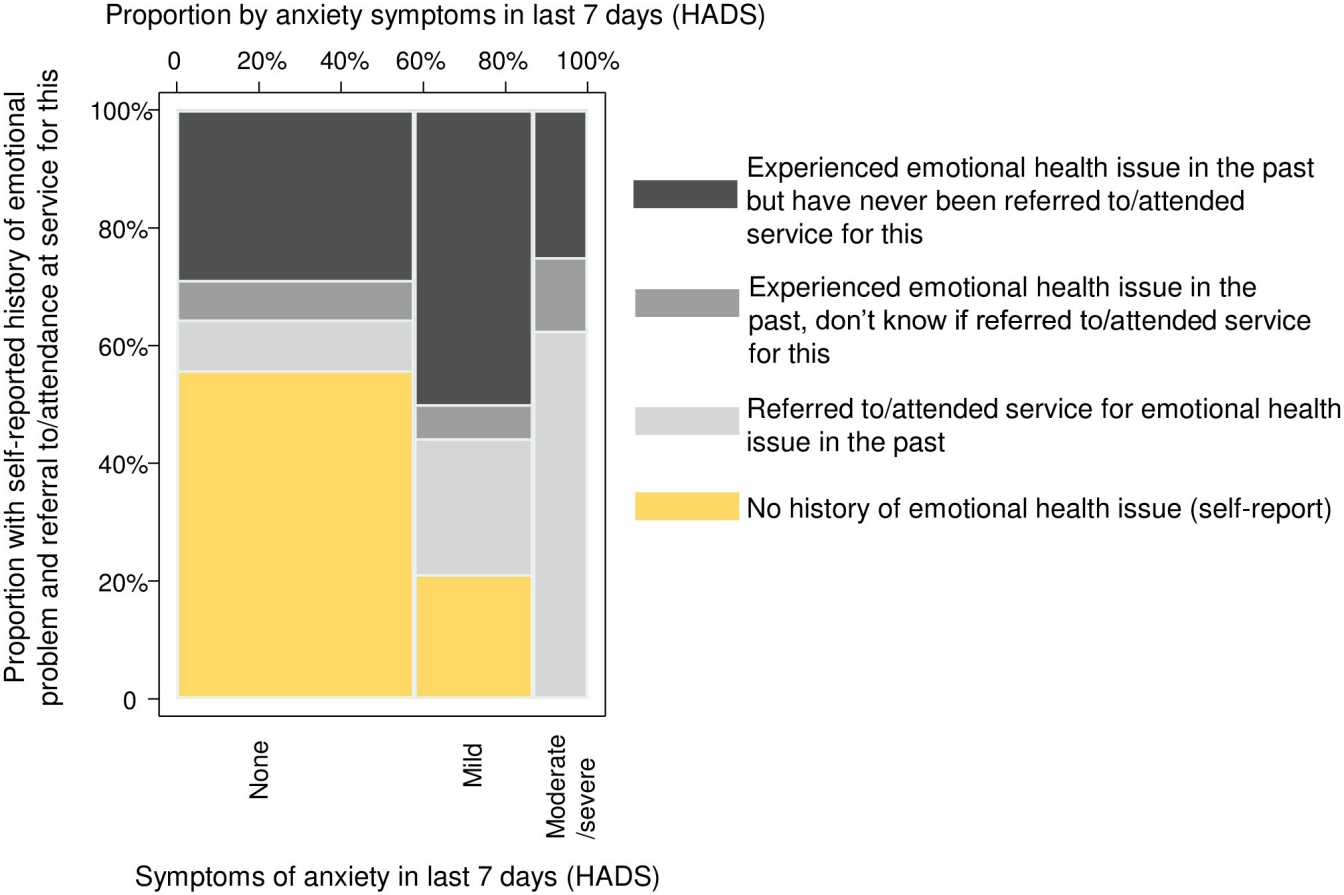
Symptoms of anxiety in the last 7 days according to Hospital Anxiety and Depression Scale by self-reported history of ever having experienced emotional problem and referral to or attendance at service for this (*n* = 180).

Fig 1 indicates symptoms of anxiety in the last 7 days according to self-reported history of emotional health issues and referral to/attendance at a service for this. Overall, 20% (36/180) reported having been referred to/received care for an emotional health issue in the past and a further 42% (75/180) reported an issue for which they had not been referred or attended for care (34%, 62/180) or were unsure (7%, 13/180). Of the remaining 38% (69/180) with no history of emotional health issue or referral to/care for this, 11 had mild anxiety symptoms at the time of the survey; none had moderate/severe symptoms according to HADS score.

Nine of the 15 respondents with moderate/severe symptoms had been referred for care in the month preceding the survey and one was currently receiving counselling. There was no difference in the proportions referred to/attending care by either mode of HIV acquisition (30% (18/61) of PHIV and 38% (20/53) in the BHIV group, *p* = 0.353) or sex (38% (23/60) of females and 28% (15/54) of males, *p* = 0.233).

### Factors associated with moderate/severe anxiety symptoms in the last 7 days

Table 3 shows factors associated with presence of moderate/severe anxiety symptoms in the last 7 days, stratified by mode of HIV acquisition. There was no association between young person’s age, or death of a parent and moderate/severe anxiety symptoms. In both PHIV and BHIV groups, young people who lived with others but had no one at home who knew the respondent’s HIV status and those reporting higher levels of HIV-related stigma were more likely to have moderate/severe anxiety symptoms. Those with higher self-esteem had lower probability of anxiety (17% reduced risk per point increase on the Rosenberg self-esteem scale overall), however this association did not remain in a minimally-adjusted model including only stigma and self-esteem (adjusted PR 0.92 95%CI 0.85–1.00, *p* = 0.06 per point increase for self-esteem and adjusted PR 1.18 95%CI 1.07–1.30, *p* = 0.001 per point increase on stigma scale). Among young people with a low CD4 count (≤350 cells/mm^3^), the proportion with moderate/severe anxiety symptoms tended to be higher among PHIV and BHIV young people, and reached statistical significance in combined analyses of PHIV and BHIV groups (Table 3, *p* = 0.035). Of the 8 young people with CD4 count ≤350 cells/mm^3^ and moderate/severe anxiety symptoms, 4 were not yet on ART (3/4 in the BHIV group).

**Table 3:** Factors associated with moderate/severe anxiety symptoms in the last 7 days.

	*BHIV group*			*PHIV group*			*Overall*	
	*n/N* (%) with anxiety in last 7 days	Unadjusted Prevalence Ratio (95% CI)	*p-*value	*n/N* (%) with anxiety in last 7 days	Unadjusted Prevalence Ratio (95% CI)	*p-*value	Unadjusted Prevalence Ratio (95% CI)	*p-*value
**Age (per increasing year)**		0.92 (0.77, 1.10)	0.357		1.08 (0.81, 1.44)	0.590	1.04 (0.95, 1.14)	0.377
**Death of parent(s)**								
No	11/69 (16)	1		2/34 (6)	1		1	
Yes	4/18 (22)	1.39 (0.50, 3.89)	0.526	7/57 (12)	2.09 (0.46, 9.56)	0.343	1.16 (0.55, 2.45)	0.694
**Do the people you currently live with know you are HIV-positive?**								
Yes, all know	3/30 (10)	1		2/55 (4)	1		1	
Only some know	4/41 (10)	0.98 (0.23, 4.08)	0.973	4/28 (14)	3.93 (0.76, 20.34)	0.103	1.97 (0.67, 5.77)	0.216
None know	3/7 (43)	4.29 (1.08, 17.06)	0.039	4/6 (67)	18.33 (4.17, 80.68)	<0.001	9.15 (3.40, 24.66)	<0.001
**Self-esteem**^a^								
Per one point increase		0.85 (0.78, 0.94)	0.001		0.81 (0.72, 0.90)	<0.001	0.83 (0.78, 0.90)	<0.001
**HIV-related stigma**^b^								
Per one unit increase		1.23 (1.08, 1.40)	0.002		1.29 (1.12, 1.48)	<0.001	1.24 (1.14, 1.34)	<0.001
**CD4 count (cells/mm**^**3**^**)**								
>350	8/62 (13)	1		7/80 (9)			1	
≤350	6/25 (24)	1.86 (0.71, 4.84)	0.204	2/8 (25)	2.86 (0.70, 11.60)	0.142	2.29 (1.06, 4.97)	0.035
**Number of home moves in the past 3 years**								
0	3/41 (7)	1		8/68 (12)				
1	6/37 (16)	2.22 (0.59, 8.30)	0.237	2/17 (12)				
≥2	6/12 (50)	6.83 (1.99, 23.48)	0.002	0/7 (0)				
**Currently smoking**								
No	6/43 (14)	1		10/83 (12)				
Yes	7/47 (15)	1.07 (0.39, 2.94)	0.900	0/9 (0)				
**Hazardous drinking or alcohol use disorder**								
No	6/63 (6)	1		10/88 (11)				
Yes	5/25 (20)	2.10 (0.70, 6.30)	0.186	0/5 (0)				
**Ever used drugs**								
No	7/76 (9)	1		10/88 (11)				
Yes	6/14 (43)	4.65 (1.83, 11.85)	0.001	0/4 (0)				
**Currently on ART**								
Yes	7/62 (11)	1		9/90 (10)				
No	8/30 (27)	2.36 (0.94, 5.93)	0.067	1/3 (33)				
**In education or employment**								
No	3/39 (8)	1		0/4 (0)				
Yes	12/54 (22)	2.89 (0.87, 9.62)	0.084	9/88 (10)				

^a^. Rosenberg Self-Esteem Scale [26], higher scores indicate higher self-esteem

^b^. abbreviated version [29], higher scores indicate more severe stigma;

Among the BHIV group, history of drug use was associated with over a four-fold increased risk of recent moderate/severe anxiety. Those with ≥2 home moves in the past three years also had increased risk compared with those with no moves. Current smoking, hazardous drinking/alcohol use disorder, being on ART and education/employment status were not associated with recent anxiety symptoms. Among 13 BHIV young people starting HIV care in the month preceding survey completion, 5 (38%) had moderate/severe anxiety symptoms vs 10/79 (13%) of those receiving HIV care for longer (*p* = 0.034). EFV use was not associated with anxiety among the 152 young people on ART (11% (6/55) in EFV group had anxiety symptoms vs 10% (10/97) among those on other regimens, *p* = 1.00) (data not shown).

## Discussion

In a sample of PHIV and BHIV young people in two regions of Ukraine, we found a 43% (95%CI 36–50%) prevalence of any anxiety symptoms and 13% (95%CI 9–19%) prevalence of moderate/severe anxiety symptoms in the last seven days, with no difference by mode of HIV acquisition, sex or age. Factors associated with moderate/ severe anxiety symptoms were higher levels of perceived stigma, lower self-esteem, having no-one at home aware of the respondent’s HIV status, low CD4 count and, among BHIV young people, illicit drug use and less stable living situation. Prevalence of recent depression symptoms was 13% (95%CI 8–18) (moderate/severe in 5%, 95%CI 3–10%); all those with moderate/severe depression symptoms also had anxiety symptoms.

The characteristics of the survey sample provide some insights into the complexities of the lived experiences of PHIV and BHIV young people in Ukraine, as well as the broader changing epidemiology of the HIV epidemic as it affects young people. In the BHIV group, 59% were female, reflecting the predominance of women among newly diagnosed 15–24 year olds (accounting for around 10% of new diagnoses nationally in 2016 while male 15–24 year olds accounted for 4% [33]), and possible gender differences in health-seeking behaviour–particularly as 20/51 of BHIV females were currently pregnant, which may have prompted HIV diagnosis and/or engagement with services. Over half of BHIV young women were mothers, mostly living with their child(ren). Notably, most of young BHIV males in our sample were MSM, a group systematically misclassified as belonging to the heterosexual risk group in official national HIV figures [34], and who are particularly vulnerable to stigma and discrimination. The preponderance of MSM among the male BHIV group in our survey may reflect a recent trend reported in integrated biobehavioural surveys of an increase in HIV prevalence among MSM in Ukraine overall, and particularly among those <25 years (from 1.9% in 2013 to 4.3% in 2015), concurrent with stable HIV prevalence among other key population groups [35]. It also demonstrates the value of computer-assisted self-interview for the collection of sensitive information on potentially stigmatised behaviours in this population. The inclusion of both PHIV and BHIV young people resulted in a diverse sample with respect to time since HIV diagnosis and experience of ART, with almost all of the PHIV group on ART (for median 9 years) vs only two-thirds of the BHIV group, most of whom had registered for HIV care within the previous year. Of note, over a quarter of the BHIV young people had a low CD4 count (≤350 cells/mm^3^) indicating late presentation to HIV services.

Despite slightly poorer psychological functioning as measured by MMQL tool in the BHIV group, anxiety prevalence was similar by mode of HIV acquisition and sex. Studies comparing mental health outcomes by mode of HIV acquisition are scarce; however two studies (both in the USA) found higher prevalence of emotional problems among BHIV young people than PHIV [13, 36] with one reporting an anxiety prevalence among BHIV similar to our survey (around 17%) [13]. Comparisons across studies are often difficult to interpret due to contextual differences which may impact on psychosocial health, within/between population differences (e.g. the proportion of BHIV young people who are PWID) and the range of tools/outcomes used. However, studies of PHIV and HIV exposed/affected youth have found broadly similar results to our survey. A recent UK study of PHIV and HIV-affected negative young people, mostly black African with median age 16 years, found a prevalence of moderate/severe anxiety symptoms using HADS of around 15–18% in both groups [10]. Meanwhile in the USA, emotional problems were reported by 17% of HIV-exposed and 12% of PHIV youth in one study [9] while another found a 25% prevalence of any mood disorder among 197 PHIV youth [37]. Among PHIV and HIV-affected negative ≤18 year olds followed up for two years in the USA IMPAACT study, anxiety screening thresholds were cumulatively met by 24% of PHIV and 37% of the control group [8], while the prevalence of any anxiety was 48–49% among PHIV and HIV exposed uninfected young people in another study [12], similar to the 43% prevalence of any anxiety symptoms in our sample.

Survey responses indicated some unmet need for mental health support, with 42% of respondents overall reporting a previous emotional health issue for which they had not been referred/attended for care. This may have included some issues not severe enough to meet referral thresholds or not disclosed to health care providers. Psychologists and/or social workers are available to young people at most HIV/AIDS centres in Ukraine [6]; however, comprehensive education and clinical training for psychologists is lacking [22] and community mental health services are very limited [22, 38]. Out-of-pocket expenses for psychiatric medication and services also impede access [39]. In 2013, national plans were developed to reform child and adolescent psychiatric services but implementation has been challenging, including a shortage of psychiatrists and psychologists with paediatric/adolescent expertise [38]. For young PLWH, community-based organisations with expertise in youth and HIV-specific issues may be an important source of support and NGO-supported community mental health services are available in some regions, although with potential issues around sustainability [22]. Overall, only 10% of young people in our survey had gained access to peer counselling and 10% had attended a support group–figures which alongside our survey results indicate unmet need for psychosocial support.

HIV-related stigma was associated with increased likelihood of anxiety symptoms here, as also reported from studies in Sweden, China and the USA, where stigma was associated with worse quality of life, negative emotions, and higher anxiety/depression scores [16, 17, 20, 40], as well as poorer adherence to ART [17]. The higher levels of stigma reported by BHIV young people across all stigma sub-scales in our study may be due to factors linked with risk of HIV acquisition and experiences of stigma (e.g. being an MSM), or the fact that BHIV young people had more commonly disclosed their HIV status to someone (71% vs 45% PHIV). Linked to this, our results indicated that young people keeping their HIV status a secret at home were more likely to report anxiety symptoms, underscoring the role of non-disclosure as a source of stress, and the importance of social support [41]. In a previous study of married/cohabiting mothers in Ukraine who were mostly in their 20s, 10% had not disclosed their HIV status to their partner [42], while a national survey in 2013 found that 82% of PLWH had negative feelings towards themselves due to HIV status [15]. Self- or anticipated stigma may be a barrier to disclosure of HIV status to others and to seeking emotional and practical support, and this may become more relevant for the PHIV group as they move away from family in the coming years and navigate disclosure to new friends and partners [43]. The association between lower self-esteem and poorer mental health has been found among PHIV young people elsewhere [10, 44] and in general population samples [45], and is a potential area for intervention; however, even after adjusting for self-esteem, felt stigma remained associated with recent moderate/severe anxiety in our study.

Of those BHIV young people included in our study, a history of drug use was associated with anxiety symptoms as has been found elsewhere [46], possibly reflecting links between drug use and adverse circumstances or stressful events [47], including the use of drugs to relieve anxiety and psychological distress, or anxiety-inducing effects of drugs themselves.

Data on mental health in the general population is extremely limited in Ukraine, and thus comparable normative data for our tools was lacking. Some of these tools have not been validated in young people; however most of our respondents were older adolescents or in their 20s and the tools were well completed, with responses indicating high levels of reliability in this population. Inclusion of BHIV and PHIV young people supports the generalisability of our findings and there is evidence that self-administered, computer-based approaches facilitate disclosure of sensitive behaviours [48]. Nevertheless, there are some limitations in interpretations. The HADS tool is a screening rather than diagnostic tool, data on access to emotional health services relied on self-report, and severity of the issues reported was uncertain. Due to the moderate size of our sample and collinearity, small numbers and missing data across some characteristics investigated, our main analyses were fitting unadjusted models stratified by mode of HIV acquisition rather than fully adjusted models. This survey was conducted among young people engaged with HIV care, which may have introduced sampling bias related to differential engagement with HIV care and willingness to take part by gender and other characteristics. In particular, young people with IDU-acquired HIV may experience specific barriers and delays to entering HIV care [49, 50]; only three completed our survey (not included in analyses), indicating that a similar study in this group would require different methodology, for example community-based sampling. Finally, improvements over time in mental health measures among PHIV or HIV-affected young people reported elsewhere [11, 12] could not be assessed in our cross-sectional study.

In conclusion, in this sample of PHIV and BHIV young people receiving HIV care in Kiev and Odessa, 13% had recent moderate/severe anxiety symptoms (in the last week), with some unmet need for mental health support identified overall. Recent anxiety symptoms were associated with lower self-esteem, higher HIV-related felt stigma and non-disclosure of HIV status at home in both PHIV and BHIV groups. Although BHIV young people comprise the majority of young PLWH [6, 33], demand for services for PHIV adolescents and young adults will increase in coming years as the PHIV group ages [3]. Future work is needed to explore how best to provide mental health support to both groups, particularly around disclosure, self-esteem and stigma.

## Supporting information

S1 File Study questionnaire in English and Russian. (PDF)
