# Supplementary material for: Anxiety symptoms and felt stigma among young people living with perinatally or behaviourally-acquired HIV in Ukraine: A cross-sectional survey
Source: PLoS One. 2019 Jan 24;14(1):e0210412. doi: 10.1371/journal.pone.0210412 (PMC6345580; doi:10.1371/journal.pone.0210412)
Supplement: S1 File — (PDF) [file pone.0210412.s001.pdf]

Acknowledgements:
Sections of this survey were developed and kindly shared by the UK's Adolescents and Adults Living with Perinatal HIV Cohort (AALPHI). For more information about AALPHI, please see http://www.ctu.mrc.ac.uk/our_research/research_areas/hiv/studies/aalphi/.

Additional acknowledgements are as follows:

- Section 2 Question 14: These eight statements are from The Pediatric HIV/AIDS Cohort Study (PHACS) study (https://phacsstudy.org/).
- Section 2, Question 21: Lu et al, AIDS Behav 2008; 12:86-94 and Feldman AIDS Behav 2013 Jan;17(1):307-18
- Section 2, Questions 22, 24, 28: AACTG Adherence Instruments (reasons for missed dose adapted); Chesney et al AIDS Care 2000; 12:3, 255-266, DOI: 10.1080/09540120050042891
- Section 2, Question 29: this is the Belief in Medicines Questionnaire (BMQ) ART version. Horne et al. International Journal of Std & Aids, 2004. **15**(1): p. 38-44.
- Section 3, Questions 1-10 form the Rosenberg self-esteem scale (Rosenberg, M. (1965). Society and the adolescent self-image. Princeton, NJ: Princeton University Press)
- Section 3, Questions 11-20 are taken from the HIV Stigma Scale (Berger et al, Res in Nurs & Health 2001, 24: 518-529), abbreviated version by Wright et al J Adolesc Health 2007, 40(1): 96-98
- Section 3, Questions 21-65 make up the Minneapolis-Manchester Quality of Life Instrument, Adolescent Form, Bhatia et al, 2002. J Clin Oncol 20:4692-4698.
- The Hospital Anxiety and Depression Scale was also included in Section 3, but is not reproduced here due to copyright considerations. It was originally published by A.S. Zigmond, R.P. Snaith, 1983 Acta Psychiatr Scand, 67 (1983), pp. 361–370. For review of its use, see Herrmann C, International experiences with the Hospital Anxiety and Depression Scale – A review of validation data and clinical results. 1997. Journal of Psychosomatic Research, 42(1) pp17-41.
- Section 4, Question 4 is from the UK's National Survey of Sexual Attitudes and Lifestyles. For more information see http://www.natsal.ac.uk/home.aspx
- Section 5, Questions 1-3. AUDIT-C screening tool, Bradley et al., AUDIT-C as a brief screen for alcohol misuse in primary care. Alcohol Clin Exp Res, 2007. **31**(7): p. 1208-17.

English: pages 2 to 33
Russian translation: pages 34 to 65

To be completed by clinician before giving the computer tablet to the young person

Today's date __/__/____

Centre Kiev ☐ Odessa ☐

Study number ____________________

- *For young people enrolled in the Ukraine Paediatric HIV Cohort, this should be their cohort study number.*
- *For all other young people, please assign a new unique identifier beginning "KYP" for Kiev and "OYP" for Odessa (see below). Keep a secure record of this identifier and use it to complete the clinical details questionnaire.*

  Kiev: KYP1, KYP2, KYP3 etc.
  Odessa: OYP1, OYP2, OYP3 etc.

Date of birth __/__/____

Sex: Male ☐ Female ☐

For their HIV care, this young person:

Has only ever been seen by a paediatrician ☐
Has only ever been seen by an adult physician ☐
Is now seen by an adult physician, following transfer from paediatric services ☐
Currently being seen by both a paediatrician and an adult physician ☐

The rest of the survey is for completion by the young person in private

Thank you for taking part in this research project.

The answers you provide in this survey are confidential, and will not be fed back to your doctor or parent /carer.

If you have any concerns about any of the topics in this survey, you can talk to your doctor or an NGO providing support – contact details of organisations are given on the information sheet, along with contact details of two local researchers who can answer questions about the study.

You can miss out any questions you do not want to answer. If you want to stop the survey before getting to the end, keep clicking "Next" to get to the end of the questionnaire, then press "Submit" and give the tablet back to the doctor. They will not be able to see any answers you have already completed.

Some questions may seem more relevant to you than others. If any questions do not quite fit with your situation, please just answer them as best you can.

Section 1: About yourself

1. Who do you live with at the moment? Select all that apply
(Please select everyone you live with – e.g. if you sometimes live with mother and sometimes with father, please select both)

Mother ☐
Father ☐
Adopted, foster or step mother ☐
Adopted, foster or step father ☐
Brother or sister ☐
Grandmother ☐
Grandfather ☐
Husband or wife ☐
Partner (boyfriend or girlfriend) ☐
My biological child /children ☐
My step child /step children ☐
Other family member(s) (e.g. aunt, uncle) ☐
Friend(s) / housemates ☐
On my own ☐
In an institution (orphanage etc) ☐
Homeless ☐
Other ☐ Specify____________________________

1a. If child /children selected from list above: how many children do you have?

1 ☐
2 ☐
3 or more ☐

1b. If >1 child: Do they all live with you?

Yes ☐
No ☐
Sometimes ☐

1c. If child/children **not** selected from list above: do you have any children that don't live with you?

Yes ☐
No ☐

1d. If yes: How many children do you have?

1 ☐
2 ☐
3 or more ☐

1e. If mother not selected from list: Is your mother alive?

Yes ☐
No ☐
Don't know ☐

If no, how old were you when she died? _________years /don't know

Do you know what the cause of death was?____

1f. If father not selected from list: Is your father alive?

Yes ☐
No ☐
Don't know ☐

If no, how old were you when he died? _________years /don't know

Do you know what the cause of death was?____

1g. If husband or wife / partner not selected from list: Do you have a partner /boyfriend or girlfriend who you don't live with?

Yes ☐
No ☐

2. How many different places have you lived in in the past three years?

Just one ☐
Two different places ☐
Three different places ☐
Four or more different places ☐

3. How old were you when you found out that you were HIV-positive?

______________years /can't remember

4. *For those not currently homeless or living alone:*
Do the people you currently live know that you are HIV-positive?

Yes they all know ☐
Only some of them know ☐
None of them know ☐

5. Who have you told that you are HIV-positive? Only include people you have told yourself. Select all that apply. If you have not told anyone, please select last option.

Parent or guardian	☐	
Husband, wife or partner (boyfriend or girlfriend)	☐	
Other family members	☐	
Friends	☐	
Employer or teacher at school /college	☐	
Other	☐	Specify____________________________
No one	☐	

6. Are you still in education (at school, college, or university)?

Yes, in full-time education	☐ Go to 6c
Yes, in part-time education	☐ Go to 6a, 6b and 6c
No - not in education anymore	☐ Go to 6a and 6b.

6a. How old were you when you left full-time education? _______ years

6b. Are you in paid employment at the moment?

Yes – employed full-time (30 hours or more per week)	☐ Go to 6c
Yes – employed part-time (less than 30 hours a week)	☐ Go to 6c
Yes – casual work / irregular hours	☐ Go to 6c
No – unemployed and looking for work	☐
No – long-term sick /disabled	☐
No – looking after home /family	☐
No – other	☐ Specify _________________

6c. How many days off work or education have you had in the last month due to sickness? ________days

SECTION 2: This section is about your experiences of HIV care and HIV medicine
As with the rest of the survey, your doctor will not see your answers.

1. How long does it normally take you to get to the HIV centre?
   - Less than 30 minutes ☐
   - From 30 minutes to one hour ☐
   - 1-2 hours ☐
   - More than 2 hours ☐

2. How long did you wait at the centre before seeing the doctor today?
   - Less than 15 minutes ☐
   - 15-30 minutes ☐
   - From 30 minutes to one hour ☐
   - 1-2 hours ☐
   - More than 2 hours ☐

3. Do you attend the HIV/AIDS centre for care, when you are asked /recommended to by your doctor?
   - Yes, always ☐
   - Most of the time ☐Go to 3a
   - Not very often ☐Go to 3a

3a. When you haven't attended the HIV/AIDS centre, why was this? Select all that apply
   - It takes too long to travel to the centre ☐
   - It costs too much to travel to the centre ☐
   - I have to wait too long at the centre to be seen ☐
   - I don't like the way that I am treated by staff ☐
   - I don't feel comfortable being in the clinic with the other patients ☐
   - I didn't want to receive medical care ☐
   - Other ☐ Specify_________

4. Does someone usually come with you when you come to see the doctor?
   - No, I come by myself ☐
   - Yes, parent or adopted /foster parent ☐
   - Yes, other family member ☐
   - Yes, friend ☐
   - Yes, social worker ☐
   - Yes, someone else ☐ Specify __________________

5. Have you or your family / carer ever needed to pay for treatment /services as part of your HIV care?
   - No ☐
   - Yes ☐ Go to 5a.
   - Don't know ☐

5a. Do you know if the payment was for something specific? (Select all that apply)

Not specific – general payment ☐
Yes – for HIV medicines ☐
Yes – for blood tests ☐
Yes – for vaccinations ☐
Yes – for equipment /supplies ☐
Yes – for costs of staying in hospital ☐
Yes – for something else ☐ Specify ______________________________
Don't know ☐

Question 6 is for children in paediatric care

6. Has anyone talked to you about you starting to be seen in adult HIV services when you are older?

Yes ☐Go to 6a and 6b.
No ☐
Don't know ☐

6a. How old were you when anyone first talked to you about this?

(Multiple choice – 11 to 18 years, or other)

6b. Were you told what age this would occur?

No ☐
Yes ☐ Specify ________________years

Questions 7-9 are for young people seeing a paediatrician and adult physician

7. How old were you when you first saw the adult physician?

(Multiple choice – 15 to 22 years or other)

8. How many times have you seen the adult physician so far?

1 ☐
2 ☐
3 or more ☐

9. Do you know when you will start seeing the adult physician only?

No ☐
Yes ☐

If yes, multiple choice 15-22 years or other

Questions 10-12 are for young people who are now being seen in adult services following transfer from paediatric services

10. At what age did you first see the adult physician?

Multiple choice – 15-22 years or other

11. When you first started to see the adult physician, did you continue to see the paediatrician as well for a period of time?

Yes, there was a period when I saw both doctors ☐
No, I did not see the paediatrician any more after seeing adult doctor ☐

12. How prepared did you feel to begin receiving your HIV care in adult services?

Very prepared ☐
Quite prepared ☐
Not very prepared ☐
Not prepared at all ☐
Don't know ☐

13. Can you think of anything that would have made you feel more prepared? Select any that apply or tell us your ideas

Knowing more about my HIV medicines ☐
Knowing more about HIV generally ☐
Getting to know the new doctor better first ☐
More opportunity to talk to other young people living with HIV ☐
Something else ☐ Specify_____________

The following questions are for all young people

14. For each of the following statements, please choose the answer that best applies to you.

I make, reschedule and cancel my own healthcare appointments

Yes, I do this ☐
No, someone else does it for me ☐

If I needed to, I could tell someone the names of all my HIV medicines

Yes, I do this ☐
No, someone else does it for me ☐

For each of my HIV medicines, I could tell someone the number of pills I take every day

Yes, I do this ☐
No, someone else does it for me ☐

I am able to name some of the possible side effects of my HIV medicines

Yes, I do this ☐
No, someone else does it for me ☐

I make my own travel arrangements to get to my clinic appointments

Yes, I do this ☐
No, someone else does it for me ☐

I tell the clinic when I need more medicines

Yes, I do this ☐
No, someone else does it for me ☐

I tell my doctor/nurse how my health has been since my last appointment

Yes, I do this ☐
No, someone else does it for me ☐

If I needed to, I could tell someone my most recent CD4 count and viral load

Yes, I do this ☐
No, someone else does it for me ☐

15. Would you recommend the HIV centre you attend to other young people with HIV?

No ☐
Yes ☐
Not sure ☐

16. Can you say why you would /would not recommend the HIV centre? (open question)

17. Which changes do you think would improve services at the HIV centre for young people? Select all that apply

More flexible opening times (e.g. evening or weekend clinic) ☐
Shorter waiting time ☐
A time when the clinic is open just for young people ☐
Help with transport costs ☐
General support from psychologist ☐
Something else ☐ Specify___________________

18. Are you currently using any of the following services (Select all that apply):

Social services ☐
Peer counselling ☐
Treatment adherence counselling ☐
Support group ☐
Other support service ☐ Specify_________________________________

19. Are you currently taking medicines for your HIV?

Yes ☐
No ☐ Go to 25

20. How often do you take HIV medication?

Once a day ☐
Twice a day ☐
Three times a day ☐

21. How would you rate your ability to take your HIV medicines as prescribed?

- Excellent ☐
- Very good ☐
- Good ☐
- Fair /OK ☐
- Poor ☐
- Very poor ☐

22. Over the last three days have you missed a dose of your HIV medication?

- Yes, today ☐
- Yes, yesterday ☐
- Yes, day before yesterday ☐
- No ☐

23. In the last month have you missed more than two days in a row of your HIV medication?

- Yes ☐
- No ☐

24. For those who report missing a dose in questions 21 or 22: What are the main reasons you have not taken your HIV medication?

- I was busy with other things ☐
- I had run out of medicine ☐
- I was away from home ☐
- I simply forgot ☐
- I felt like the medicine was toxic or harmful ☐
- I have doubts about taking medicines due to my religious beliefs ☐
- I wanted to avoid side effects (feeling sick etc) ☐
- I thought the medicine wasn’t helping ☐
- I felt well /didn’t think I needed it ☐
- Taking medicine is difficult with school /work hours ☐
- I was asleep when I was supposed to be taking the medicine ☐
- I did not want other people to know I was taking medicine ☐
- I was unwell ☐
- My parent /caregiver forgot to give it to me ☐
- I was too tired ☐
- I couldn’t deal with it that day ☐
- I had too much medicine to take ☐
- My routine was different from normal (weekend etc) ☐
- I am fed up taking medicine ☐
- Other (Specify) ☐Specify____________

25. Have you ever missed a dose of medicine because the HIV/AIDS centre ran out? (Had a stockout)

- Yes ☐
- No ☐

Those currently on ART stop here

26. For those not on ART currently: Have you taken HIV medicines in the past?

Yes ☐ Continue
No ☐End of this section

27. Who decided that you should stop your HIV medicine?

You ☐
Doctor ☐
Parent /carer ☐

28. Select the main reasons that you stopped:

The medicine was not available anymore at the HIV/AIDS centre ☐
I would run out of medicine ☐
I was away from home a lot ☐
I would forgot ☐
Felt like the medicine was toxic or harmful ☐
Doubts about taking medicines due to my religious beliefs ☐
Wanted to avoid side effects (feeling sick etc) ☐
Thought the medicine wasn't helping ☐
I was well /didn't need it ☐
Taking medicine is difficult with school /work hours ☐
Sleeping when I was supposed to be taking the medicine ☐
Did not want other people to know I was taking medicine ☐
I was unwell ☐
My parent /caregiver would forget to give it to me ☐
I was too tired ☐
Couldn't deal with it that day ☐
I had too much medicine to take ☐
My routine was different from normal (weekend etc) ☐
I was fed up taking medicine ☐
Other (Specify) ☐Specify___________

29. We would like to ask you about your personal views about HIV medication. (Also sometimes called HAART or HIV or combination therapy.)
These are statements other people have made about their HIV medication.
Please show how much you agree or disagree with them by ticking the box.
There are no right or wrong answers. We are interested in your personal views.

My health, at present depends on HIV medication

Strongly agree ☐
Agree ☐
Uncertain ☐
Disagree ☐
Strongly disagree ☐

Having to take HIV medication worries me

Strongly agree ☐
Agree ☐
Uncertain ☐
Disagree ☐
Strongly disagree ☐

My life would be impossible without HIV medication

Strongly agree ☐
Agree ☐
Uncertain ☐
Disagree ☐
Strongly disagree ☐

I sometimes worry about the long-term effects of HIV medication

Strongly agree ☐
Agree ☐
Uncertain ☐
Disagree ☐
Strongly disagree ☐

Without HIV medication I would be very ill

Strongly agree ☐
Agree ☐
Uncertain ☐
Disagree ☐
Strongly disagree ☐

HIV medication is a mystery to me

Strongly agree ☐
Agree ☐
Uncertain ☐
Disagree ☐
Strongly disagree ☐

My health in the future will depend on HIV medication

Strongly agree ☐
Agree ☐
Uncertain ☐
Disagree ☐
Strongly disagree ☐

HIV medication disrupts my life

Strongly agree ☐
Agree ☐
Uncertain ☐
Disagree ☐
Strongly disagree ☐

I sometimes worry about becoming too dependent on HIV medication

Strongly agree ☐
Agree ☐
Uncertain ☐
Disagree ☐
Strongly disagree ☐

HIV medication keeps my HIV under control

Strongly agree ☐
Agree ☐
Uncertain ☐
Disagree ☐
Strongly disagree ☐

HIV medication gives me unpleasant side effects

Strongly agree ☐
Agree ☐
Uncertain ☐
Disagree ☐
Strongly disagree ☐

Using HIV medication is embarrassing

Strongly agree ☐
Agree ☐
Uncertain ☐
Disagree ☐
Strongly disagree ☐

Missing this medication for a day won't matter in the long run

Strongly agree ☐
Agree ☐
Uncertain ☐
Disagree ☐
Strongly disagree ☐

HIV medication is my best hope for the future

Strongly agree ☐
Agree ☐
Uncertain ☐
Disagree ☐
Strongly disagree ☐

I am unlikely to get a bad side effect from HIV medication in the next month

Strongly agree ☐
Agree ☐
Uncertain ☐
Disagree ☐
Strongly disagree ☐

Taking HIV medication has been worse than expected

Strongly agree ☐
Agree ☐
Uncertain ☐
Disagree ☐
Strongly disagree ☐

I have received enough information about HIV medication

Strongly agree ☐
Agree ☐
Uncertain ☐
Disagree ☐
Strongly disagree ☐

HIV medication keeps me alive

Strongly agree ☐
Agree ☐
Uncertain ☐
Disagree ☐
Strongly disagree ☐

The taste of HIV medication makes me feel unwell

Strongly agree ☐
Agree ☐
Uncertain ☐
Disagree ☐
Strongly disagree ☐

Section 3: This section is about how you think about yourself. Please let us know whether you agree or disagree with each statement.
There are no right or wrong answers, we are interested in your honest opinion – how you think or feel

1. I feel that I am a person of worth, at least on an equal plane with others
   - Strongly agree ☐
   - Agree ☐
   - Disagree ☐
   - Strongly disagree ☐

2. I feel that I have a number of good qualities
   - Strongly agree ☐
   - Agree ☐
   - Disagree ☐
   - Strongly disagree ☐

3. All in all, I am inclined to feel that I am a failure.
   - Strongly agree ☐
   - Agree ☐
   - Disagree ☐
   - Strongly disagree ☐

4. I am able to do things as well as most other people
   - Strongly agree ☐
   - Agree ☐
   - Disagree ☐
   - Strongly disagree ☐

5. I feel I do not have much to be proud of
   - Strongly agree ☐
   - Agree ☐
   - Disagree ☐
   - Strongly disagree ☐

6. I take a positive attitude toward myself
   - Strongly agree ☐
   - Agree ☐
   - Disagree ☐
   - Strongly disagree ☐

7. On the whole, I am satisfied with myself
   - Strongly agree ☐
   - Agree ☐
   - Disagree ☐
   - Strongly disagree ☐

8. I wish I could have more respect for myself

- Strongly agree ☐
- Agree ☐
- Disagree ☐
- Strongly disagree ☐

9. I certainly feel useless at times

- Strongly agree ☐
- Agree ☐
- Disagree ☐
- Strongly disagree ☐

10. At times I think I am no good at all

- Strongly agree ☐
- Agree ☐
- Disagree ☐
- Strongly disagree ☐

11. I have been hurt by how people reacted to learning I have HIV

- Strongly agree ☐
- Agree ☐
- Disagree ☐
- Strongly disagree ☐
- I've never told anyone ☐

12. I am very careful who I tell that I have HIV

- Strongly agree ☐
- Agree ☐
- Disagree ☐
- Strongly disagree ☐

13. I feel that I am not as good a person as others because I have HIV

- Strongly agree ☐
- Agree ☐
- Disagree ☐
- Strongly disagree ☐

14. Most people think that a person with HIV is disgusting

- Strongly agree ☐
- Agree ☐
- Disagree ☐
- Strongly disagree ☐

15. I have stopped socialising with some people because of their reactions to my having HIV

- Strongly agree ☐
- Agree ☐

Disagree ☐
Strongly disagree ☐
I've never told those I socialise with ☐

16. I worry that people who know I have HIV will tell others
Strongly agree ☐
Agree ☐
Disagree ☐
Strongly disagree ☐

17. Having HIV makes me feel unclean
Strongly agree ☐
Agree ☐
Disagree ☐
Strongly disagree ☐

18. Most people with HIV are rejected when others find out
Strongly agree ☐
Agree ☐
Disagree ☐
Strongly disagree ☐

19. I have lost friends by telling them I have HIV
Strongly agree ☐
Agree ☐
Disagree ☐
Strongly disagree ☐
I've never told anyone ☐

20. Having HIV makes me feel that I'm a bad person
Strongly agree ☐
Agree ☐
Disagree ☐
Strongly disagree ☐

We are interested in finding out what young people like yourself think about their own health and how it might affect the things they do at school, with friends and so on. This includes asking about the activities you do and how you feel about different aspects of daily life.
There are no right or wrong answers, but it is important that you say what you really think. Your answers will not be seen by anyone you know, including your parents/carers.

Please mark your answers by putting a tick in the box next to the answer you choose.

The following statements are about sports and activities you may do:

21. How often does your health stop you from taking part in sports such as football, swimming, riding a bike or similar activities?

    Always ☐
    Almost always ☐
    Sometimes ☐
    Almost never ☐
    Never ☐

22. How often is it difficult to keep up with others of the same age when taking part in sports or games?

    Always ☐
    Almost always ☐
    Sometimes ☐
    Almost never ☐
    Never ☐

23. How often do you feel tired during the day?

    Always ☐
    Almost always ☐
    Sometimes ☐
    Almost never ☐
    Never ☐

24. How often do you feel strong and healthy?

    Always ☐
    Almost always ☐
    Sometimes ☐
    Almost never ☐
    Never ☐

25. I need time to rest during the day

    Strongly agree ☐
    Agree ☐
    Neither agree nor disagree ☐
    Disagree ☐
    Strongly disagree ☐

26. I have a lot of energy

Strongly agree ☐
Agree ☐
Neither agree nor disagree ☐
Disagree ☐
Strongly disagree ☐

27. I have a lot of energy for running or sports

Strongly agree ☐
Agree ☐
Neither agree nor disagree ☐
Disagree ☐
Strongly disagree ☐

28. I cannot do many activities because of my health

Strongly agree ☐
Agree ☐
Neither agree nor disagree ☐
Disagree ☐
Strongly disagree ☐

29. In games and sports, I would rather watch than take part

Strongly agree ☐
Agree ☐
Neither agree nor disagree ☐
Disagree ☐
Strongly disagree ☐

How often do you feel:

30. Sad

Always ☐
Almost always ☐
Sometimes ☐
Almost never ☐
Never ☐

31. Angry

Always ☐
Almost always ☐
Sometimes ☐
Almost never ☐
Never ☐

32. Lonely

Always ☐
Almost always ☐
Sometimes ☐
Almost never ☐
Never ☐

33. Frightened

Always ☐
Almost always ☐
Sometimes ☐
Almost never ☐
Never ☐

34. Anxious and nervous

Always ☐
Almost always ☐
Sometimes ☐
Almost never ☐
Never ☐

35. Worried about things in general

Always ☐
Almost always ☐
Sometimes ☐
Almost never ☐
Never ☐

36. Worried about your health

Always ☐
Almost always ☐
Sometimes ☐
Almost never ☐
Never ☐

37. Worried about dying

Always ☐
Almost always ☐
Sometimes ☐
Almost never ☐
Never ☐

38. Not as good as other people

Always ☐
Almost always ☐
Sometimes ☐
Almost never ☐
Never ☐

The following statements are about your physical appearance

39. I am happy with my weight

Strongly agree ☐
Agree ☐
Neither agree nor disagree ☐
Disagree ☐
Strongly disagree ☐

40. I am happy with the way I look

Strongly agree ☐
Agree ☐
Neither agree nor disagree ☐
Disagree ☐
Strongly disagree ☐

41. I am happy with my body development at the moment

Strongly agree ☐
Agree ☐
Neither agree nor disagree ☐
Disagree ☐
Strongly disagree ☐

42. I like my body the way it is

Strongly agree ☐
Agree ☐
Neither agree nor disagree ☐
Disagree ☐
Strongly disagree ☐

43. When others look at me they think I am poorly developed

Strongly agree ☐
Agree ☐
Neither agree nor disagree ☐
Disagree ☐
Strongly disagree ☐

44. I am uncomfortable with the way my body is developing

Strongly agree ☐
Agree ☐
Neither agree nor disagree ☐
Disagree ☐
Strongly disagree ☐

The following statements are about how you get on with others:

45. I find it difficult to make friends

Strongly agree ☐
Agree ☐
Neither agree nor disagree ☐
Disagree ☐
Strongly disagree ☐

46. I feel left out in groups of people my own age

Strongly agree ☐
Agree ☐
Neither agree nor disagree ☐
Disagree ☐
Strongly disagree ☐

47. People like to be with me

Strongly agree ☐
Agree ☐
Neither agree nor disagree ☐
Disagree ☐
Strongly disagree ☐

48. I have a lot in common with my friends

Strongly agree ☐
Agree ☐
Neither agree nor disagree ☐
Disagree ☐
Strongly disagree ☐

49. I get on well with people my own age

Strongly agree ☐
Agree ☐
Neither agree nor disagree ☐
Disagree ☐
Strongly disagree ☐

50. I have many close friends

Strongly agree ☐
Agree ☐
Neither agree nor disagree ☐
Disagree ☐
Strongly disagree ☐

51. I have similar hobbies and interests to other people of the same age

Strongly agree ☐
Agree ☐
Neither agree nor disagree ☐
Disagree ☐
Strongly disagree ☐

52. Being with other people makes me happy

Strongly agree ☐
Agree ☐
Neither agree nor disagree ☐
Disagree ☐
Strongly disagree ☐

53. I find it easy to have an intimate relationship

Strongly agree ☐
Agree ☐
Neither agree nor disagree ☐
Disagree ☐
Strongly disagree ☐

54. I am confident when I am with people of the opposite sex

Strongly agree ☐
Agree ☐
Neither agree nor disagree ☐
Disagree ☐
Strongly disagree ☐

The following statements are about how you get on at school, college or work:

55. I have difficulty concentrating at work /school /college

Strongly agree ☐
Agree ☐
Neither agree nor disagree ☐
Disagree ☐
Strongly disagree ☐

56. I have difficulty concentrating at other times (eg playing cards, computer games, reading)

Strongly agree ☐
Agree ☐
Neither agree nor disagree ☐
Disagree ☐
Strongly disagree ☐

57. Homework or study is hard for me

Strongly agree ☐
Agree ☐
Neither agree nor disagree ☐
Disagree ☐
Strongly disagree ☐

58. I need more help with school/college work than others in my class

Strongly agree ☐
Agree ☐
Neither agree nor disagree ☐
Disagree ☐
Strongly disagree ☐

59. I have difficulty remembering things at school / college / work

Strongly agree ☐
Agree ☐
Neither agree nor disagree ☐
Disagree ☐
Strongly disagree ☐

60. I have difficulty with reading and writing

Strongly agree ☐
Agree ☐
Neither agree nor disagree ☐
Disagree ☐
Strongly disagree ☐

61. I have difficulty with maths and calculations

Strongly agree ☐
Agree ☐
Neither agree nor disagree ☐
Disagree ☐
Strongly disagree ☐

62. I have more difficulty with my school/ college /work compared with others

- Strongly agree ☐
- Agree ☐
- Neither agree nor disagree ☐
- Disagree ☐
- Strongly disagree ☐

Please tell us how much you agree with the following statements:

63. I am happy with the way things are

- Strongly agree ☐
- Agree ☐
- Neither agree nor disagree ☐
- Disagree ☐
- Strongly disagree ☐

64. I am happy with life in general

- Strongly agree ☐
- Agree ☐
- Neither agree nor disagree ☐
- Disagree ☐
- Strongly disagree ☐

65. In general I am satisfied with my current life situation

- Strongly agree ☐
- Agree ☐
- Neither agree nor disagree ☐
- Disagree ☐
- Strongly disagree ☐

At this point in the survey, the Hospital Anxiety and Depression Scale is included but is not reproduced here because it is copyrighted.
The Hospital Anxiety and Depression Scale questions are supplemented by the questions below:

1. Have you or anyone else ever thought that you had low mood (depression), fears and worries (anxiety), aggression or anger, or any other emotional health issues?
    - No ☐
    - Yes ☐

2. Have you ever been referred to or attended a service for low mood (depression), fears and worries (anxiety), aggression or anger, or any other emotional health issues?

No ☐
Yes – for low mood (depression) ☐
Yes – for fears and worries (anxiety) ☐
Yes – for self harm ☐
Yes – for anger or aggression ☐
Yes – for other emotional health issue ☐ Specify ____________________________
Don't know ☐

2a. If yes to any service in question 2: When was this?

In last month ☐
In last year ☐
More than a year ago ☐

3. Have you ever been given any of the following for emotional health issues? Select all that apply

Antidepressants ☐ If selected, being given currently? ☐
One to one counselling ☐ If selected, being given currently? ☐
Stay in hospital ☐

Section 4: Sexual and reproductive health

This section includes questions you may find sensitive or personal. Your answers are important to us as they will allow us to understand young people's needs. If you don't want to answer a question, please select "don't want to answer" or miss it out. All of your answers are confidential and will not be shared with your doctor or parent/carer.

1. Have you ever had vaginal, anal or oral sex? (*Vaginal sex is penis inside the vagina, anal sex is penis inside the bottom, oral sex is penis or vagina in the mouth).*

Yes ☐ Go to 3.
No ☐ Go to question 2, then skip rest of this section and go to section 5.
Don't want to answer ☐ Go to the next section (section 5).

2. If you have not had vaginal, anal or oral sex, can you give the reason(s) why? Select all that apply:

Not ready to have sex / don't want to ☐
Religious beliefs ☐
Partner doesn't want to ☐
Fear of HIV transmission ☐
Family pressure ☐
Not had the opportunity ☐
Other ☐ Specify ______________________________

3. Which types of sex have you **<u>ever</u>** had (select all that apply)

Vaginal ☐
Anal ☐
Oral ☐
Don't want to answer ☐

3a. Which types of sex have you had in the **last year**? (select all that apply)

- Vaginal ☐
- Anal ☐
- Oral ☐
- None ☐
- Don't want to answer ☐

3b. Which types of sex have you had in the **last four weeks**? (select all that apply)

- Vaginal ☐
- Anal ☐
- Oral ☐
- None ☐
- Don't want to answer ☐

4. Which of these descriptions applies best to you and the person you most recently had sex with?
   - It was someone I didn't know ☐
   - We had recently met ☐
   - We had known each other for a while, but were not in a steady relationship ☐
   - We used to be in a steady relationship, but were not at that time ☐
   - We were in a steady relationship at that time ☐
   - We were living together as a couple / married at that time ☐
   - Other ☐
   - Don't want to answer ☐

5. In total, how many partners have you **ever** had vaginal, anal or oral sex with?

   1 ☐ 2 ☐ 3-4 ☐ 5-9 ☐ 10 or more ☐

5a. How many sexual partners have you had **in the last year**?

0 ☐ 1 ☐ 2 ☐ 3-4 ☐ 5-9 ☐ 10 or more ☐

6. Have your sexual partners been:
   - Men only ☐
   - Women only ☐
   - Both men and women ☐ Go to 6a and 6b

6a. How many opposite-sex partners have you ever had vaginal, anal or oral sex with? *(opposite-sex partners are female partners if you are male, or male partners if you are female)*

1 ☐ 2 ☐ 3-4 ☐ 5-9 ☐ 10 or more ☐

6b. How many opposite-sex partners have you had sex with in the last year?

0 ☐ 1 ☐ 2 ☐ 3-4 ☐ 5-9 ☐ 10 or more ☐

7. How many times have you had vaginal, anal or oral sex in the last four weeks?

   _____________times

(question hidden if no sexual partners in the last four weeks)

8. Have you ever had sex in return for money, drugs or some other gift?
   - No ☐
   - Yes ☐
   - Don't want to answer ☐

9. Have you ever told a sexual partner that you are HIV-positive?
   - No ☐
   - Yes ☐
   - Can't remember ☐

9a. Have you told your current /most recent sexual partner that you were HIV-positive?
   - No ☐
   - Yes ☐
   - Can't remember ☐

10. What is the HIV status of your current /most recent sexual partner?
   - I don't know ☐
   - HIV-positive ☐
   - HIV-negative ☐

11. In the past year have you ever had vaginal or anal sex without a condom?
   - Yes - I have had sex without a condom ☐ Go to 11a
   - No ☐

11a. How many people have you had vaginal or anal sex with, without a condom, in the last year?
   1 ☐ 2 ☐ 3-4 ☐ 5-9 ☐ 10 or more ☐

12. Have you been tested for sexually transmitted infections in the last year?
   - Yes ☐ Go to 12a.
   - No ☐
   - Don't know ☐
   - Don't want to answer ☐

12a. Where were you tested?
   - HIV/AIDS centre ☐
   - Somewhere else (e.g. polyclinic, sexual health /family planning clinic) ☐

13. Have you had a sexually transmitted infection in the last year?
   - No ☐
   - Yes ☐ Go to 13a.
   - Don't want to answer ☐

14. Which one(s)? Select all that apply

- Chlamydia ☐
- Gonorrhoea ☐
- Syphilis ☐
- Genital herpes ☐
- Trichmoniasis ☐
- Bacterial vaginosis (BV) ☐
- Genital warts ☐
- Pelvic inflammatory disease (PID) ☐
- Can't remember name ☐
- Other ☐ Specify___________________________________

15. How important is it for you to avoid pregnancy now (in you or your partner)?

- Very important ☐
- Quite important ☐
- Not at all important ☐

16. Have you and the person /people you have had sex with in the last 12 months been using any family planning method? (Select all that apply)

- Condoms ☐ Go to 16a
- Oral contraceptive pill ☐
- Intra-uterine device ☐
- Injectable contraceptive ☐
- Contraceptive implant ☐
- Contraceptive patch ☐
- Withdrawal method ☐
- Sterilised ☐
- Other ☐ Specify_____________________________
- None ☐

16a. Where do you buy or receive condoms? Select all that apply

- Drug store, convenience or grocery store ☐
- Vending machine ☐
- Bars or clubs ☐
- HIV/AIDS centre ☐
- Community medical /health clinic ☐
- Other community organisation ☐
- Sexual partner has them ☐
- Family ☐
- Other ☐ Specify____________________________

17. Do you have access to condoms free-of-charge if you want them?

- Yes, as many as I need ☐
- Yes, but only sometimes ☐
- No ☐

18. For women only: have you ever had a pregnancy termination? If so, how many?

Never had a termination ☐
Yes – 1 ☐
Yes - 2 ☐
Yes - 3 or more ☐

Section 5: This section is about smoking and use of alcohol and drugs

1. How often do you have a drink containing alcohol?

Never ☐ Go to 4.
Monthly or less ☐
2-4 times a month ☐
2-3 times a week ☐
4 or more times a week ☐

For the next two questions, consider one drink to be a bottle of beer, a small glass of wine, or a shot of vodka or other spirits.

2. How many drinks containing alcohol do you have on a typical day when drinking?

1 or 2 ☐ 3 or 4 ☐ 5 or 6 ☐ 7, 8 or 9 ☐ 10 or more ☐

3. How often do you have six or more drinks on one occasion?

Never ☐
Less than monthly ☐
Monthly ☐
Weekly ☐
Daily or almost daily ☐

4. Have you ever smoked cigarettes or tobacco?

No ☐
Yes ☐ Go to 4a

4a. Do you currently smoke cigarettes or tobacco?

No ☐
Yes ☐ Go to 4b

4b. Do you smoke every day or occasionally?

Every day ☐ Specify how many a day ______________________cigarettes
Occasionally ☐

5. Have you ever used drugs recreationally?

No ☐
Yes ☐

5a. Do you currently live with anyone who uses street drugs?

No	☐ Skip the rest of this section
Yes	☐ Go to 5b
Don't want to answer	☐ Skip the rest of this section

5b. Who do you live with who uses street drugs? Select all that apply

Friend /flatmate	☐
Partner (boyfriend, girlfriend etc)	☐
Parent	☐
Other family member	☐
Don't want to answer	☐

6. How old were you when you first started using any drugs?

_____________years

7. Have you ever considered yourself to be addicted to street drugs?

No	☐
Yes	☐

8. What kind of drugs have you used in the past? Select all that apply

Marijuana (cannabis, hashish)	☐
Heroin	☐
Homemade opiates (shirka)	☐
Amphetamines (vint, jeff etc)	☐
Ecstasy	☐
Desomorphine (Crocodile)	☐
Methedrone (bath salts)	☐
Street methadone	☐
Benzodiazepines	☐
Other	☐ Specify______________________________

9. Have you ever, or are you currently, injecting drugs?

Yes, in the past	☐ Go to 9a, 9b
Yes, currently	☐ Go to 9a, 9b
No, I've never injected	☐

9a. How old were you when you first injected?

_________________years

9b. Who did the first injection for you?

Drug dealer	☐
Sibling	☐
Friend	☐
Partner	☐
No one – I did it myself	☐
Someone else	☐ Specify ________________

11. Do you currently use street drugs?

- No ☐
- Yes ☐ Go to 11a
- Don't want to answer ☐

11a. What kind of drugs do you use currently?

- Marijuana (cannabis, hashish) ☐
- Heroin ☐
- Homemade opiates (shirka) ☐
- Amphetamines (vint, jeff etc) ☐
- Ecstasy ☐
- Desomorphine (Crocodile) ☐
- Methedrone (bath salts) ☐
- Street methadone ☐
- Benzodiazepines ☐
- Other ☐ Specify________________________________

12. Have you ever been registered with addiction services?

- No ☐
- Yes ☐

13. Have you ever accessed harm reduction services for drug users?

- No ☐
- Yes ☐ Go to 14a

13a. Which services have you used or were you offered in harm reduction services?

- Clean needles /syringes ☐
- Condoms ☐
- Contraception counselling ☐
- Pregnancy testing ☐
- HIV testing ☐
- Testing for other sexually transmitted infections ☐
- Information and referral for HIV ☐
- Information and referral for methadone /buprenorphine ☐
- Hepatitis B vaccination ☐
- Other ☐ Specify __________________

14. Have you ever received treatment for drug use? Select all that apply

- Abstinence (detox / rehab) ☐
- Methadone /buprenorphine substitution ☐ Go to 14a, 14b, 14c, 14d
- None of these ☐

14a. Do you currently receive methadone or buprenorphine as part of a drug treatment programme?

- No ☐
- Yes ☐

14b. How often do you receive methadone /buprenorphine, or did you in the past?

Daily ☐
Every 2 days ☐
Other ☐ Specify ______________________

14c. Where do you receive methadone /buprenorphine, or did you in the past?

Addiction clinic ☐
HIV/AIDS centre ☐
Paid buprenorphine outlet ☐
Other ☐ Specify ____________________________

14d. How long did it usually take you to get to this place, from home?

_______hours _______ min

15. Did you find any of the questions in this survey unclear or difficult to understand?

No ☐
Yes, one or two questions ☐ Go to 15a
Yes, three or more questions ☐ Go to 15a

15a. If yes to Question 15: Did you ask anyone for help to understand the questions?

No ☐
Yes ☐

Thank you very much for completing this survey.
If you have any concerns about any of the topics in this survey or any worries, or if there is anything you would like to discuss, please tell your doctor. There is a list of organisations providing information and support to young people living with HIV on your information sheet.

We would like to tell young people living with HIV about the results of this research. What do you think is the best way of doing this?

Leaflet and poster at HIV/AIDS centre ☐
Website ☐
Discussion group for young people ☐
Other –please tell us your ideas___________________________________

Опрос молодежи в Центрах профилактики и борьбы с ВИЧ/СПИДом в Киеве и Одессе

Заполняется врачом перед выдачей планшета юноше или девушке

Сегодняшняя дата __/__/____

Центр Киев ☐ Одесса ☐

Идентификационный номер участника исследования ____________________

- *Если опрашиваемый молодой человек (юноша или девушка) включен в Украинское педиатрическое когортное исследование ВИЧ-инфекции, следует указать идентификационный номер, присвоенный ему (ей) в этом когортном исследовании.*
- *Всем остальным юношам и девушкам следует присваивать уникальные идентификаторы, начинающиеся с букв «KYP» для Киева и «OYP» для Одессы (см. ниже). Храните записи о соответствии идентификаторов личным данным в защищенном месте и используйте этот идентификатор для заполнения клинического опросника.*
  Киев: KYP1, KYP2, KYP3 и т. д.
  Одесса: OYP1, OYP2, OYP3 и т. д.

Дата рождения __/__/____

Пол: Мужской ☐ Женский ☐

Этот юноша или девушка наблюдается по поводу ВИЧ-инфекции:
- Наблюдается только у инфекциониста-педиатра ☐
- Наблюдается только у инфекциониста взрослой сети ☐
- В настоящее время наблюдается у инфекциониста взрослой сети, к которому переведен(-а) от инфекциониста-педиатра ☐
- В настоящее время наблюдается одновременно у инфекциониста взрослой сети и у инфекциониста педиатрической сети ☐

Остальная часть опроса предназначена для заполнения молодым человеком в приватной обстановке.

Благодарим вас за участие в этом научном исследовании.

Ответы, которые вы дадите на вопросы этого опросника, будут сохранены в тайне и не будут переданы вашему врачу или родителям (опекунам).

Если у вас возникнут любые вопросы по поводу любого раздела данного опросника, вы можете задать их своему врачу или сотруднику благотворительной организации, предоставляющей поддержку ВИЧ-инфицированным – контактная информация этих организаций указана на информационном листке; кроме того, на этом листке указана контактная информация двух исследователей, которые могут ответить на ваши вопросы об этом исследовании.

Вы можете пропустить любые вопросы, на которые не хотите отвечать. Если вы захотите прекратить отвечать на вопросы, не дойдя до конца опросника, нажмите «Далее», чтобы перейти на следующий экран, на котором вы еще не отвечали на вопросы, и затем отдайте планшет врачу. Врач не сможет просмотреть ваши ответы.

Некоторые вопросы касаются вас в большей степени, чем другие. Если какой-либо вопрос относится к вам не в полной мере, пожалуйста, постарайтесь выбрать наиболее подходящий ответ.

Раздел 1. Расскажите о себе

1. С кем вы сейчас живете? Выберите все подходящие ответы
   (Пожалуйста, укажите всех, с кем вы живете – например, если вы живете то с матерью, то с отцом, укажите обоих родителей)

Мать	☐
Отец	☐
Приемная мать или мачеха	☐
Приемный отец или отчим	☐
Брат или сестра	☐
Бабушка	☐
Дедушка	☐
Муж или жена	☐
Партнер или партнерша	☐
Родной (биологический) ребенок (дети)	☐
Приемный ребенок (дети)	☐
Другой родственник или родственники (например, тетя или дядя)	☐
Друг (друзья) / соседи по жилью (общежитию)	☐
Живу самостоятельно	☐
Живу в учреждении (приюте, детском доме и т. д.)	☐
Бездомный(-ая)	☐
Другое	☐ Укажите_____________________________

1a. Если вы указали, что живете с детьми: сколько у вас детей?

1	☐
2	☐
3+	☐

1b. Если у вас >1 ребенка: Все ваши дети живут с вами?

Нет ☐
Да ☐
Иногда ☐

1c. Если вы **не** указали, что живете с детьми: есть ли у вас дети, с которыми вы не живете вместе?

Нет ☐
Да ☐

1d. Если да: сколько у вас детей?

1 ☐
2 ☐
3+ ☐

1e. Если вы не живете с матерью: ваша мать жива?

Нет ☐
Да ☐
Не знаю ☐

Если вашей матери нет в живых, то сколько вам было лет, когда ваша мать умерла?
_________лет / не знаю

Знаете ли вы, что было причиной ее смерти?_______________________________________

1f. Если вы не живете с отцом: ваш отец жив?

Нет ☐
Да ☐
Не знаю ☐

Если вашего отца нет в живых, то сколько вам было лет, когда ваш отец умер?
_________лет / не знаю

Знаете ли вы, что было причиной его смерти?_______________________________________

1g. Если вы не живете с мужем (или женой) или с партнером (партнершей):
У вас есть муж (жена) или партнер (девушка или парень), с которым (которой) вы не живете вместе?

Нет ☐
Да ☐

2. Сколько мест жительства вы сменили за последние три года?

Я жил(а) только в одном месте ☐
Два разных места ☐
Три разных места ☐
Четыре и более разных мест ☐

3. В каком возрасте вы узнали о том, что ВИЧ-инфицированы?

_____________лет /не помню

4. *Вопрос для тех, кто в настоящее время не бездомный и не живет один:*
Люди, с которыми вы сейчас живете, знают о том, что вы инфицированы ВИЧ?

Да, они все знают об этом ☐
Только некоторые знают об этом ☐
Никто не знает об этом ☐

5. Кому вы сказали о том, что ВИЧ-инфицированы? Укажите только тех, кому вы сами сказали об этом. Если вы никому не говорили об этом, выберите последний вариант ответа.

Мать или Отец ☐
Муж или жена, Партнер или партнерша ☐
Другой родственник (родственники) ☐
Друг (друзья) ☐
Работодатель, Учитель в школе / преподаватель в учебном заведении ☐
Другое ☐
Я никому не говорил(-а) ☐

6. Вы еще учитесь (в школе, училище, техникуме или институте)?

Да, на очном (дневном) отделении ☐ Перейдите к вопросу 6с
Да, на заочном отделении (или учусь время от времени) ☐ Перейдите к вопросам 6а, 6b и 6с
Нет, я уже не учусь ☐ Перейдите к вопросам 6а и 6b.

6а. В каком возрасте вы прекратили очное (дневное, постоянное) обучение? _______ лет

6b. У вас сейчас есть оплачиваемая работа?

Да, полная занятость (30 часов и более в неделю) ☐ Перейдите к вопросу 6с
Да, неполная занятость (меньше 30 часов в неделю) ☐ Перейдите к вопросу 6с
Да – случайные/нерегулярные подработки ☐ Перейдите к вопросу 6с
Нет – у меня нет работы, но я ее сейчас ищу ☐
Нет – я не могу работать из-за хронического заболевания / инвалидности ☐
Нет – я домохозяйка/домохозяин, ухаживаю за родственниками ☐
Нет – другое ☐ Укажите _________________

6с. Сколько учебных или рабочих дней в прошлом месяце вы пропустили из-за болезни?
________дней

Раздел 2. Этот раздел посвящен вашему опыту посещения Центра ВИЧ/СПИДа и лечения ВИЧ-инфекции
Ваш врач не увидит ваши ответы на эти и любые другие вопросы данного опросника.

1. Сколько времени у вас обычно занимает дорога до Центра ВИЧ/СПИДа?
   - менее 30 минут ☐
   - от 30 минут до часа ☐
   - Один -два часа ☐
   - Более двух часов ☐

2. Сколько вы сегодня ждали приема у врача в Центре?
   - Менее 15 минут ☐
   - 15-30 минут ☐
   - от 30 минут до часа ☐
   - Один -два часа ☐
   - Более двух часов ☐

3. Вы приходите на приемы в Центр так часто, как вам назначено (рекомендовано) врачом?
   - Да, прихожу на все назначенные приемы ☐
   - Прихожу на большую часть назначенных приемов ☐Перейдите к вопросу 3а
   - Прихожу в Центр редко ☐Перейдите к вопросу 3а

3а. По какой причине вы пропускаете приемы в Центре ВИЧ/СПИДа? Выберите все подходящие ответы
   - До Центра слишком долго добираться ☐
   - Дорога до Центра обходится очень дорого ☐
   - В Центре слишком долго приходится ждать приема ☐
   - Мне не нравится отношение ко мне персонала Центра ☐
   - Мне неловко находиться в Центре вместе с другим пациентами ☐
   - Другое ☐ Укажите __________
   - я не хотел получать медицинские услуги ☐

4. Вас сопровождает кто-то на приемы к врачу?
   - Нет, я хожу один (одна) ☐
   - Да, родитель или опекун (приемный родитель) ☐
   - Да, другой родственник ☐
   - Да, друг ☐
   - Да, социальный работник ☐
   - Да, кто-то еще ☐ Укажите ___________________

5. Когда-нибудь вам или вашим родственникам (опекунам) приходилось платить за медицинские услуги, предоставленные вам в рамках лечения ВИЧ-инфекции?
   - Нет ☐
   - Да ☐ Перейдите к вопросу 5а.
   - Не знаю ☐

5a. Вам пришлось заплатить за что-то конкретное? (Выберите все подходящие ответы)

Платили за медицинскую помощь в целом ☐
Да – за лекарства для лечения ВИЧ-инфекции ☐
Да – за анализы крови ☐
Да – за вакцинацию ☐
Да – за расходные материалы/инструменты ☐
Да – за пребывание в больнице ☐
Да – за что-то другое ☐ Укажите ______________________________
Не знаю ☐

Вопрос 6 относится к детям, наблюдающимся у педиатра

6. Кто-нибудь говорил с вами о том, что вы, когда станете старше, будете наблюдаться у инфекциониста взрослой сети?

Да ☐Перейдите к вопросам 6a и 6b.
Нет ☐
Не знаю ☐

6a. Сколько вам было лет, когда вам об этом сказали в первый раз?

_______________лет

6b. Вам сказали, в каком возрасте вы перейдете под наблюдение инфекциониста взрослой сети?

Нет ☐
Да ☐ Укажите ________________лет

Вопросы 7-9 относятся к молодым людям, посещающим педиатра и врача взрослой сети

7. В каком возрасте вы впервые посетили инфекциониста взрослой сети?

_______________лет

8. Сколько раз вы уже посетили инфекциониста взрослой сети? (Дайте примерный ответ, если вы не помните точное число посещений)

______________________раз

9. Вы знаете, в каком возрасте вы будете посещать только инфекциониста взрослой сети?

Нет ☐
Да ☐ Укажите_________________ лет

Вопросы 10-12 относятся к молодым людям, которые ранее посещали педиатра, а теперь посещают инфекциониста взрослой сети

10. В каком возрасте вы впервые посетили врача взрослой сети? _______________лет

11. Когда вы начали посещать врача взрослой сети, продолжали ли вы в течение некоторого времени посещать также педиатра?

Да, в течение некоторого времени я наблюдался(-ась) у обоих врачей ☐
Нет, я больше не ходил(-а) к педиатру после того, как стал(-а) ходить к врачу взрослой сети ☐

12. Насколько вы были готовы лечиться у инфекциониста взрослой сети?

- Совершенно готов(-а) ☐
- Достаточно готов(-а) ☐
- Не совсем готов(-а) ☐
- Совсем не готов(-а) ☐
- Не знаю ☐

13. Как можно было бы лучше подготовить вас к лечению у врача взрослой сети? Выберите все подходящие ответы или напишите свои предложения

- Больше знаний о лекарствах, назначенных мне для лечения ВИЧ-инфекции ☐
- Больше знаний о ВИЧ-инфекции ☐
- Предварительное знакомство с новым врачом ☐
- Больше возможностей общения с молодыми людьми, живущими с ВИЧ-инфекцией ☐
- Что-то еще ☐ Укажите_____________

Следующие вопросы предназначены для всех молодых людей

14. Для каждого утверждения выберите наиболее подходящий ответ.

Я сам(-а) записываюсь / переношу на другое время / отменяю свои приемы у врача

- Да, я это делаю ☐
- Нет, это за меня делает другой человек ☐

Если нужно, я могу перечислить названия всех своих лекарств от ВИЧ-инфекции

- Да, я это могу ☐
- Нет, это может сделать только другой человек ☐

Я могу сказать, сколько таблеток каждого лекарства от ВИЧ-инфекции я принимаю каждый день

- Да, я это могу ☐
- Нет, это может сделать только другой человек ☐

Я могу назвать некоторые возможные побочные эффекты своих лекарств от ВИЧ-инфекции

- Да, я это могу ☐
- Нет, это может сделать только другой человек ☐

Я сам(-а) организую свою поездку в Центр на назначенный прием к врачу

- Да, я это делаю ☐
- Нет, это за меня делает другой человек ☐

Я сообщаю сотрудникам клиники о том, что у меня заканчиваются лекарства

- Да, я это делаю ☐
- Нет, это за меня делает другой человек ☐

Я сам(-а) рассказываю врачу/медсестре о своем самочувствии со дня предыдущего визита

- Да, я это делаю ☐
- Нет, это за меня делает другой человек ☐

Если нужно, я могу сообщить свои последние показатели количества лимфоцитов CD4 и вирусной нагрузки

Да, я это делаю ☐

Нет, это может сделать только другой человек ☐

15. Вы бы посоветовали другим молодым людям с ВИЧ-инфекцией лечиться в Центре ВИЧ/СПИДа, который посещаете вы сами?

Нет ☐

Да ☐

Не уверен(-а) ☐

16. Можете ли вы объяснить, почему вы советуете или не советуете обращаться в этот Центр ВИЧ/СПИДа? (открытый вопрос, подразумевает развернутый ответ)

17. Как вы думаете, каким образом можно улучшить медицинское обслуживание молодых людей в Центре ВИЧ/СПИДа? Выберите все подходящие ответы

Сделать график работы Центра удобнее для посетителей (например, возможность посещений в вечернее время или в выходные дни) ☐

Сократить время ожидания приема ☐

Выделить отдельные часы приема только для молодых людей ☐

Возмещать расходы на транспорт ☐

Помощь психолога ☐

Что-то еще ☐ Укажите_____________

18. Пользуетесь ли вы сейчас каким-либо услугами из перечисленных ниже? (Выберите все подходящие ответы)

Помощь социального работника ☐

помощь консультантов "равный равному” ☐

Консультирование по поводу соблюдения режима приема препаратов ☐

Участие в группах поддержки ☐

Другая поддержка ☐ Укажите________________________________

19. Вы сейчас принимаете лекарства для лечения ВИЧ-инфекции (антиретровирусную терапию)?

Нет ☐ Перейдите к вопросу 25

Да ☐ Продолжайте отвечать

20. Как часто вы принимаете лекарства для лечения ВИЧ-инфекции?

Один раз в сутки ☐

Два раза в сутки ☐

Три раза в сутки ☐

21. Как бы вы оценили свои способности принимать лекарства для лечения ВИЧ-инфекции в соответствии с указаниями врача?

Отлично ☐

Очень хорошо ☐

Хорошо ☐

Нормально/терпимо ☐

Плохо ☐

Очень плохо ☐

22. Пропускали ли вы прием лекарств для лечения ВИЧ-инфекции в течение последних трех дней?

Сегодня ☐
Вчера ☐
Позавчера ☐
Нет ☐

23. Пропускали ли вы прием лекарств для лечения ВИЧ-инфекции больше двух дней подряд в течение последнего месяца?

Да ☐
Нет ☐

24. Если вы указали, что пропускали приемы лекарств в вопросах 21 или 22: почему вы пропускали прием лекарств для лечения ВИЧ-инфекции?

У меня были другие дела ☐
У меня закончились лекарства ☐
Я был(-а) далеко от дома ☐
Я просто забыл(-а) ☐
От лекарств я чувствую себя только хуже ☐
Из-за моих религиозных убеждений я сомневаюсь в том, что мне следует принимать лекарства ☐
Я не хочу испытывать побочные эффекты (недомогание, тошноту и т. д.) ☐
Я думаю, что лекарства не помогают ☐
Я чувствую себя хорошо / я думаю, что мне не нужны лекарства ☐
Мне трудно принимать препараты, когда я в школе или на работе ☐
Я проспал(-а) время приема препарата ☐
Я не хочу, чтобы другие люди знали о том, что я принимаю лекарства ☐
Я себя плохо чувствовал(-а) ☐
Мой родитель / опекун забыл(-а) дать мне лекарства ☐
Я слишком устал(-а) ☐
Я не смог(-ла) справиться с этим в тот день ☐
Мне нужно было принимать слишком много таблеток ☐
Мой распорядок дня отличался от обычного (например, был выходной) ☐
Мне надоело принимать лекарства ☐
Другое (укажите) ☐Укажите____________

25. Вы когда-нибудь пропускали прием препаратов из-за того, что в Центре не было ваших препаратов (закончились)?

Нет ☐
Да ☐

Если вы сейчас получаете антиретровирусную терапию, то перейдите к вопросам следующего раздела

26. Вопросы для тех, кто сейчас не получает антиретровирусные препараты: вы принимали препараты для лечения ВИЧ-инфекции раньше?

Нет ☐Вы закончили отвечать на вопросы этого раздела
Да ☐ Продолжайте отвечать на вопросы этого раздела

27. Кто решил, что вы должны прекратить принимать препараты для лечения ВИЧ-инфекции?

Вы сами ☐
Врач ☐
Родитель/опекун ☐

28. Укажите основные причины, из-за которых вы прекратили принимать антиретровирусные лекарства:

В Центре ВИЧ/СПИДа больше не выдаются мои лекарства ☐
У меня закончились лекарства ☐
Я был(-а) далеко от дома в течение длительного времени ☐
Я забывал(-а) принимать лекарства ☐
От лекарств я чувствовала себя только хуже ☐
Из-за моих религиозных убеждений я решил(-а), что мне не следует принимать лекарства ☐
Я не хотел(-а) испытывать побочные эффекты (недомогание, тошноту и т. д.) ☐
Я думал(-а), что лекарства мне не помогают ☐
Я чувствовал(-а) себя хорошо / я думал(-а), что мне не нужны лекарства ☐
Мне было трудно принимать препараты, когда я был(-а) в школе или на работе ☐
Я проспал(-а) время приема препарата ☐
Я не хотел(-а), чтобы другие люди знали о том, что я принимал(-а) лекарства ☐
Я себя плохо чувствовал(-а) ☐
Мой родитель / опекун забывал(-а) дать мне лекарства ☐
Я слишком устал(-а) ☐
Я не мог(-ла) справиться с необходимостью принимать лекарства ☐
Мне нужно было принимать слишком много таблеток ☐
Мой распорядок дня отличался от обычного (например, был выходной) ☐
Мне надоело принимать лекарства ☐
Другое (укажите) ☐Укажите____________

29. Нам бы хотелось узнать ваше мнение об антиретровирусных препаратах. Далее приведены утверждения других людей о своих антиретровирусных препаратах. Пожалуйста, укажите, в какой степени вы согласны или не согласны с этими утверждениями, поставив галочку напротив вашего ответа. Здесь нет правильных или неправильных ответов. Нам интересно именно ваше мнение.

- Мое здоровье сейчас зависит от приема антиретровирусных препаратов

Полностью согласен(-на) ☐
Согласен(-на) ☐
Не уверен(-а) в ответе ☐
Не согласен(-на) ☐
Категорически не согласен(-на) ☐

- Необходимость приема антиретровирусных препаратов осложняет мне жизнь

Полностью согласен(-на) ☐

Согласен(-на) ☐
Не уверен(-а) в ответе ☐
Не согласен(-на) ☐
Категорически не согласен(-на) ☐

- Я бы не выжил(-а) без антиретровирусных препаратов

Полностью согласен(-на) ☐
Согласен(-на) ☐
Не уверен(-а) в ответе ☐
Не согласен(-на) ☐
Категорически не согласен(-на) ☐

- Я иногда задумываюсь о том, как долго будут действовать препараты и какое действие они оказывают на мой организм

Полностью согласен(-на) ☐
Согласен(-на) ☐
Не уверен(-а) в ответе ☐
Не согласен(-на) ☐
Категорически не согласен(-на) ☐

- Без антиретровирусных препаратов я бы тяжело болел(-а)

Полностью согласен(-на) ☐
Согласен(-на) ☐
Не уверен(-а) в ответе ☐
Не согласен(-на) ☐
Категорически не согласен(-на) ☐

- Я ничего толком не знаю об антиретровирусных препаратах, для меня это лечение непонятно

Полностью согласен(-на) ☐
Согласен(-на) ☐
Не уверен(-а) в ответе ☐
Не согласен(-на) ☐
Категорически не согласен(-на) ☐

- Мое здоровье в будущем зависит от антиретровирусных препаратов

Полностью согласен(-на) ☐
Согласен(-на) ☐
Не уверен(-а) в ответе ☐
Не согласен(-на) ☐
Категорически не согласен(-на) ☐

- Антиретровирусные препараты разрушают мою жизнь

Полностью согласен(-на) ☐
Согласен(-на) ☐
Не уверен(-а) в ответе ☐
Не согласен(-на) ☐
Категорически не согласен(-на) ☐

- Я иногда задумываюсь о том, что у меня может возникнуть зависимость от антиретровирусных препаратов

Полностью согласен(-на) ☐
Согласен(-на) ☐
Не уверен(-а) в ответе ☐
Не согласен(-на) ☐
Категорически не согласен(-на) ☐

- Антиретровирусные препараты сдерживают развитие ВИЧ-инфекции

Полностью согласен(-на) ☐
Согласен(-на) ☐
Не уверен(-а) в ответе ☐
Не согласен(-на) ☐
Категорически не согласен(-на) ☐

- От антиретровирусных препаратов у меня возникают неприятные побочные эффекты

Полностью согласен(-на) ☐
Согласен(-на) ☐
Не уверен(-а) в ответе ☐
Не согласен(-на) ☐
Категорически не согласен(-на) ☐

- Мне стыдно или неловко принимать антиретровирусные препараты

Полностью согласен(-на) ☐
Согласен(-на) ☐
Не уверен(-а) в ответе ☐
Не согласен(-на) ☐
Категорически не согласен(-на) ☐

- Можно без последствий пропустить прием антиретровирусных препаратов в течение дня, если принимаешь их долго

Полностью согласен(-на) ☐
Согласен(-на) ☐
Не уверен(-а) в ответе ☐
Не согласен(-на) ☐
Категорически не согласен(-на) ☐

- Антиретровирусные препараты – моя надежда на будущее

Полностью согласен(-на) ☐
Согласен(-на) ☐
Не уверен(-а) в ответе ☐
Не согласен(-на) ☐
Категорически не согласен(-на) ☐

- У меня вряд ли будут побочные эффекты от приема антиретровирусных препаратов в ближайшем месяце

Полностью согласен(-на) ☐
Согласен(-на) ☐

Не уверен(-а) в ответе ☐
Не согласен(-на) ☐
Категорически не согласен(-на) ☐

- Принимать антиретровирусные препараты оказалось намного труднее, чем я ожидал(-а)

Полностью согласен(-на) ☐
Согласен(-на) ☐
Не уверен(-а) в ответе ☐
Не согласен(-на) ☐
Категорически не согласен(-на) ☐

- Я получил(а) достаточно информации об антиретровирусных препаратах

Полностью согласен(-на) ☐
Согласен(-на) ☐
Не уверен(-а) в ответе ☐
Не согласен(-на) ☐
Категорически не согласен(-на) ☐

- Благодаря антиретровирусным препаратам я все еще жив(-а)

Полностью согласен(-на) ☐
Согласен(-на) ☐
Не уверен(-а) в ответе ☐
Не согласен(-на) ☐
Категорически не согласен(-на) ☐

Я плохо себя чувствую из-за отвратительного вкуса антиретровирусных препаратов

Полностью согласен(-на) ☐
Согласен(-на) ☐
Не уверен(-а) в ответе ☐
Не согласен(-на) ☐
Категорически не согласен(-на) ☐

Раздел 3. В этом разделе содержатся вопросы о вашем отношении к себе. Пожалуйста, укажите, в какой степени вы согласны или не согласны с каждым утверждением.
Здесь нет правильных или неправильных ответов; нам интересны ваши честные ответы – что вы думаете или чувствуете

1. Я ощущаю себя достойным человеком, по крайней мере, не хуже остальных
   Полностью согласен(-на) ☐
   Согласен(-на) ☐
   Не согласен(-на) ☐
   Категорически не согласен(-на) ☐

2. Я считаю, что у меня есть ряд хороших черт
   - Полностью согласен(-на) ☐
   - Согласен(-на) ☐
   - Не согласен(-на) ☐
   - Категорически не согласен(-на) ☐

3. Я склонен(-на) считать себя неудачником(-цей)
   - Полностью согласен(-на) ☐
   - Согласен(-на) ☐
   - Не согласен(-на) ☐
   - Категорически не согласен(-на) ☐

4. Я способен(-на) делать многие вещи не хуже, чем большинство людей
   - Полностью согласен(-на) ☐
   - Согласен(-на) ☐
   - Не согласен(-на) ☐
   - Категорически не согласен(-на) ☐

5. Мне кажется, что мне особо нечем гордиться
   - Полностью согласен(-на) ☐
   - Согласен(-на) ☐
   - Не согласен(-на) ☐
   - Категорически не согласен(-на) ☐

6. Я хорошо отношусь к себе
   - Полностью согласен(-на) ☐
   - Согласен(-на) ☐
   - Не согласен(-на) ☐
   - Категорически не согласен(-на) ☐

7. В общем и целом я доволен(-на) собой
   - Полностью согласен(-на) ☐
   - Согласен(-на) ☐
   - Не согласен(-на) ☐
   - Категорически не согласен(-на) ☐

8. Мне бы хотелось больше уважать себя
   - Полностью согласен(-на) ☐
   - Согласен(-на) ☐
   - Не согласен(-на) ☐
   - Категорически не согласен(-на) ☐

9. Иногда я отчетливо ощущаю собственную бесполезность
   - Полностью согласен(-на) ☐
   - Согласен(-на) ☐
   - Не согласен(-на) ☐
   - Категорически не согласен(-на) ☐

10. Иногда мне кажется, что я ни на что не гожусь
   - Полностью согласен(-на) ☐
   - Согласен(-на) ☐
   - Не согласен(-на) ☐
   - Категорически не согласен(-на) ☐

11. Меня обижает то, как реагируют люди, узнавая, что я инфицирован(-а) ВИЧ
   - Полностью согласен(-на) ☐
   - Согласен(-на) ☐
   - Не согласен(-на) ☐
   - Категорически не согласен(-на) ☐

12. Я сообщаю о том, что инфицирован(-а) ВИЧ очень осторожно и только определенным людям
   - Полностью согласен(-на) ☐
   - Согласен(-на) ☐
   - Не согласен(-на) ☐
   - Категорически не согласен(-на) ☐

13. Я чувствую себя хуже остальных из-за того, что ВИЧ-инфицирован(-а)
   - Полностью согласен(-на) ☐
   - Согласен(-на) ☐
   - Не согласен(-на) ☐
   - Категорически не согласен(-на) ☐

14. Большинство людей испытывают отвращение к ВИЧ-инфицированным
   - Полностью согласен(-на) ☐
   - Согласен(-на) ☐
   - Не согласен(-на) ☐
   - Категорически не согласен(-на) ☐

15. Я прекратил(-а) общаться с некоторыми людьми из-за их реакции на то, что я ВИЧ-инфицирован(-а)
   - Полностью согласен(-на) ☐
   - Согласен(-на) ☐
   - Не согласен(-на) ☐
   - Категорически не согласен(-на) ☐
   - В своем кругу общения я никому не говорил(-а) о том, что инфицирован(-а) ☐

16. Я беспокоюсь о том, что люди, знающие о том, что у меня ВИЧ-инфекция, расскажут об этом другим
   - Полностью согласен(-на) ☐
   - Согласен(-на) ☐
   - Не согласен(-на) ☐
   - Категорически не согласен(-на) ☐

17. Из-за ВИЧ-инфекции я чувствую на себе клеймо
   - Полностью согласен(-на) ☐
   - Согласен(-на) ☐
   - Не согласен(-на) ☐
   - Категорически не согласен(-на) ☐

18. Большинство людей отвергают ВИЧ-инфицированных
   - Полностью согласен(-на) ☐
   - Согласен(-на) ☐
   - Не согласен(-на) ☐
   - Категорически не согласен(-на) ☐

19. Я потерял(-а) друзей, когда рассказала им о том, что инфицирован(-а)
   - Полностью согласен(-на) ☐
   - Согласен(-на) ☐
   - Не согласен(-на) ☐
   - Категорически не согласен(-на) ☐

20. Из-за ВИЧ-инфекции я чувствую себя плохим человеком
   - Полностью согласен(-на) ☐
   - Согласен(-на) ☐
   - Не согласен(-на) ☐
   - Категорически не согласен(-на) ☐

Нам интересно знать, что молодые люди, такие как вы, думают о собственном здоровье и как ВИЧ-инфекция влияет на их обучение в школе, общение с друзьями и т. д. Поэтому мы задаем вопросы о разных видах деятельности и о разных сторонах вашей повседневной жизни.
Здесь нет правильных или неправильных ответов, но нам очень важно, чтобы вы отвечали честно. Никто из тех, кто вас знает лично, не увидит ваши ответы, включая ваших родителей/опекунов.

Заполняйте опросник, отмечая выбранные вами ответы галочкой.

Следующие утверждения касаются занятий спортом и других видов активной деятельности:

21. Как часто из-за плохого самочувствия вы не могли заниматься спортом или играть в подвижные игры, например, плавать, играть в футбол, кататься на велосипеде и т. д.?
   - Всегда ☐
   - Почти всегда ☐
   - Иногда ☐
   - Почти никогда ☐
   - Никогда ☐

22. Как часто вам было трудно не отставать от своих ровесников в спорте или в подвижных играх?
   - Всегда ☐
   - Почти всегда ☐
   - Иногда ☐
   - Почти никогда ☐
   - Никогда ☐

23. Как часто вы чувствуете усталость на протяжении дня?
Всегда ☐
Почти всегда ☐
Иногда ☐
Почти никогда ☐
Никогда ☐

24. Как часто вы чувствуете себя здоровым и сильным?
Всегда ☐
Почти всегда ☐
Иногда ☐
Почти никогда ☐
Никогда ☐

25. Мне нужно отдыхать в течение дня.

Полностью согласен(-на) ☐
Согласен(-на) ☐
Ни то, ни другое ☐
Не согласен(-на) ☐
Категорически не согласен(-на) ☐

26. Я полон(-на) энергии
Полностью согласен(-на) ☐
Согласен(-на) ☐
Ни то, ни другое ☐
Не согласен(-на) ☐
Категорически не согласен(-на) ☐

27. У меня много сил на занятия спортом (например, бегом)
Полностью согласен(-на) ☐
Согласен(-на) ☐
Ни то, ни другое ☐
Не согласен(-на) ☐
Категорически не согласен(-на) ☐

28. Мое здоровье не позволяет мне заниматься многими видами спорта или играть в некоторые подвижные игры
Полностью согласен(-на) ☐
Согласен(-на) ☐
Ни то, ни другое ☐
Не согласен(-на) ☐
Категорически не согласен(-на) ☐

29. Я смотрю на занимающихся спортом и играющих в подвижные игры со стороны, но не участвую
Полностью согласен(-на) ☐
Согласен(-на) ☐
Ни то, ни другое ☐
Не согласен(-на) ☐
Категорически не согласен(-на) ☐

Как часто вы

30. грустите, печалитесь

    Всегда ☐
    Почти всегда ☐
    Иногда ☐
    Почти никогда ☐
    Никогда ☐

31. сердитесь, злитесь
    Всегда ☐
    Почти всегда ☐
    Иногда ☐
    Почти никогда ☐
    Никогда ☐

32. чувствуете себя одиноким(-ой)
    Всегда ☐
    Почти всегда ☐
    Иногда ☐
    Почти никогда ☐
    Никогда ☐

33. боитесь
    Всегда ☐
    Почти всегда ☐
    Иногда ☐
    Почти никогда ☐
    Никогда ☐

34. тревожитесь, нервничаете
    Всегда ☐
    Почти всегда ☐
    Иногда ☐
    Почти никогда ☐
    Никогда ☐

35. волнуетесь о разных вещах
    Всегда ☐
    Почти всегда ☐
    Иногда ☐
    Почти никогда ☐
    Никогда ☐

36. беспокоитесь о своем здоровье
    Всегда ☐
    Почти всегда ☐
    Иногда ☐
    Почти никогда ☐
    Никогда ☐

37. думаете о смерти
   - Всегда ☐
   - Почти всегда ☐
   - Иногда ☐
   - Почти никогда ☐
   - Никогда ☐

38. чувствуете себя никчемным, хуже остальных
   - Всегда ☐
   - Почти всегда ☐
   - Иногда ☐
   - Почти никогда ☐
   - Никогда ☐

Следующие утверждения касаются вашего внешности

39. Я доволен(-на) своим весом

   - Полностью согласен(-на) ☐
   - Согласен(-на) ☐
   - Ни то, ни другое ☐
   - Не согласен(-на) ☐
   - Категорически не согласен(-на) ☐

40. Мне нравится, как я выгляжу
   - Полностью согласен(-на) ☐
   - Согласен(-на) ☐
   - Ни то, ни другое ☐
   - Не согласен(-на) ☐
   - Категорически не согласен(-на) ☐

41. Сейчас я доволен(-на) своим телосложением
   - Полностью согласен(-на) ☐
   - Согласен(-на) ☐
   - Ни то, ни другое ☐
   - Не согласен(-на) ☐
   - Категорически не согласен(-на) ☐

42. Мне нравится мое тело таким, какое оно есть
   - Полностью согласен(-на) ☐
   - Согласен(-на) ☐
   - Ни то, ни другое ☐
   - Не согласен(-на) ☐
   - Категорически не согласен(-на) ☐

43. Когда другие смотрят на меня, они думают, что я плохо сложен(-а)
Полностью согласен(-на) ☐
Согласен(-на) ☐
Ни то, ни другое ☐
Не согласен(-на) ☐
Категорически не согласен(-на) ☐

44. Мне не нравится, как изменяется (формируется) мое тело
Полностью согласен(-на) ☐
Согласен(-на) ☐
Ни то, ни другое ☐
Не согласен(-на) ☐
Категорически не согласен(-на) ☐

Следующие утверждения касаются вашего общения с другими людьми

45. Мне тяжело заводить друзей
Полностью согласен(-на) ☐
Согласен(-на) ☐
Ни то, ни другое ☐
Не согласен(-на) ☐
Категорически не согласен(-на) ☐

46. Я чувствую себя лишним в компании ровесников
Полностью согласен(-на) ☐
Согласен(-на) ☐
Ни то, ни другое ☐
Не согласен(-на) ☐
Категорически не согласен(-на) ☐

47. Люди тянутся ко мне
Полностью согласен(-на) ☐
Согласен(-на) ☐
Ни то, ни другое ☐
Не согласен(-на) ☐
Категорически не согласен(-на) ☐

48. У меня много общего с моими друзьями
Полностью согласен(-на) ☐
Согласен(-на) ☐
Ни то, ни другое ☐
Не согласен(-на) ☐
Категорически не согласен(-на) ☐

49. Я легко нахожу общий язык с ровесниками
Полностью согласен(-на) ☐
Согласен(-на) ☐
Ни то, ни другое ☐
Не согласен(-на) ☐
Категорически не согласен(-на) ☐

50. У меня много близких друзей
- Полностью согласен(-на) ☐
- Согласен(-на) ☐
- Ни то, ни другое ☐
- Не согласен(-на) ☐
- Категорически не согласен(-на) ☐

51. У меня такие же увлечения и интересы, как и у моих ровесников
- Полностью согласен(-на) ☐
- Согласен(-на) ☐
- Ни то, ни другое ☐
- Не согласен(-на) ☐
- Категорически не согласен(-на) ☐

52. Мне нравится быть в компании других людей
- Полностью согласен(-на) ☐
- Согласен(-на) ☐
- Ни то, ни другое ☐
- Не согласен(-на) ☐
- Категорически не согласен(-на) ☐

53. Я легко вступаю в интимные отношения

- Полностью согласен(-на) ☐
- Согласен(-на) ☐
- Ни то, ни другое ☐
- Не согласен(-на) ☐
- Категорически не согласен(-на) ☐

54. Я уверенно общаюсь с людьми противоположного пола
- Полностью согласен(-на) ☐
- Согласен(-на) ☐
- Ни то, ни другое ☐
- Не согласен(-на) ☐
- Категорически не согласен(-на) ☐

Следующие утверждения касаются вашей учебы или работы

55. Мне трудно сосредоточиться на работе или учебе
- Полностью согласен(-на) ☐
- Согласен(-на) ☐
- Ни то, ни другое ☐
- Не согласен(-на) ☐
- Категорически не согласен(-на) ☐

56. Мне трудно сосредоточиться на других видах деятельности (например, при игре в карты, при игре в компьютерные игры, при чтении)
- Полностью согласен(-на) ☐
- Согласен(-на) ☐
- Ни то, ни другое ☐
- Не согласен(-на) ☐
- Категорически не согласен(-на) ☐

57. Мне трудно выполнять домашнее задание или изучать что-либо
Полностью согласен(-на) ☐
Согласен(-на) ☐
Ни то, ни другое ☐
Не согласен(-на) ☐
Категорически не согласен(-на) ☐

58. Мне нужно больше помощи в учебе, чем другим ученикам в моем классе
Полностью согласен(-на) ☐
Согласен(-на) ☐
Ни то, ни другое ☐
Не согласен(-на) ☐
Категорически не согласен(-на) ☐

59. Мне трудно запоминать разные вещи в школе или на работе
Полностью согласен(-на) ☐
Согласен(-на) ☐
Ни то, ни другое ☐
Не согласен(-на) ☐
Категорически не согласен(-на) ☐

60. Мне трудно читать и писать
Полностью согласен(-на) ☐
Согласен(-на) ☐
Ни то, ни другое ☐
Не согласен(-на) ☐
Категорически не согласен(-на) ☐

61. Мне трудно заниматься математикой и вычислениями
Полностью согласен(-на) ☐
Согласен(-на) ☐
Ни то, ни другое ☐
Не согласен(-на) ☐
Категорически не согласен(-на) ☐

62. Мне сложнее учиться или работать по сравнению с другими

Пожалуйста, укажите, в какой степи вы согласны со следующими утверждениями.

63. Мне нравится, как у меня идут дела
Полностью согласен(-на) ☐
Согласен(-на) ☐
Ни то, ни другое ☐
Не согласен(-на) ☐
Категорически не согласен(-на) ☐

64. В целом я доволен(-на) своей жизнью
Полностью согласен(-на) ☐
Согласен(-на) ☐
Ни то, ни другое ☐
Не согласен(-на) ☐
Категорически не согласен(-на) ☐

65. В целом я доволен текущей жизненной ситуацией

Полностью согласен(-на) ☐
Согласен(-на) ☐
Ни то, ни другое ☐
Не согласен(-на) ☐
Категорически не согласен(-на) ☐

Сюда будет вставлена Госпитальная шкала тревоги и депрессии

1. Замечали ли вы или кто-то из ваших близких, что у вас подавленное настроение (депрессия), страхи или нервозность (тревога), агрессия или гнев, либо другое эмоциональное расстройство?

Нет ☐
Да ☐

2. Рекомендовали ли вам когда-либо обратиться к специалисту или получали ли вы медицинскую помощь по поводу подавленного настроения (депрессии), страхов или нервозности (тревоги), агрессии или гнева, либо другого эмоционального расстройства?

Нет ☐
Да – по поводу подавленного настроения (депрессии) ☐
Да – по поводу страхов и нервозности (тревоги) ☐
Да – по поводу нанесения себе вреда ☐
Да – по поводу гнева или агрессии ☐
Да – по поводу другого эмоционального расстройства ☐ Укажите ________________
Не знаю ☐

2а. Если вы ответили «да» на вопрос 2, то когда это было?

В прошлом месяце ☐
В прошлом году ☐
Больше года назад ☐

3. Вы получали какие-либо из перечисленных ниже видов помощи по поводу эмоционального расстройства? Выберите все подходящие ответы

Антидепрессанты ☐ Если да, то принимаете ли вы их сейчас? ☐
Личное консультирование ☐ Если да, то посещаете ли вы специалиста сейчас? ☐
Лежали в больнице ☐

Раздел 4. Сексуальное и репродуктивное здоровье

В этом разделе содержатся вопросы, которые вы можете посчитать слишком личными и интимными. Ваши ответы очень важны для нас, поскольку они помогут нам лучше понять потребности молодых людей. Если вы не хотите отвечать на какой-либо вопрос, выберите вариант «предпочту не отвечать» или просто пропустите его. Все ваши ответы сохранятся в тайне и не будут показаны вашему врачу или родителям/опекунам.

1. Вы когда-нибудь занимались вагинальным, анальным или оральным сексом? (*Вагинальный секс — когда пенис вводится во влагалище, анальный секс — когда пенис вводится в анус, а оральный секс — когда пенис вводится в рот или когда рот или язык касается половых губ или влагалища*)

Да ☐ Перейдите к вопросу 3
Нет ☐ Перейдите к вопросу 2, затем пропустите остальные вопросы этого раздела и перейдите к разделу 5.
Предпочту не отвечать ☐ Перейдите к следующему разделу (разделу 5).

2. Если вы еще не занимались вагинальным, анальным или оральным сексом, то почему? Выберите все подходящие ответы

Еще не готов(-а) / не хочу заниматься сексом ☐
Из-за религиозных убеждений ☐
Мой партнер или моя партнерша не хочет ☐
Страх заразить партнера или партнершу ВИЧ ☐
Давление со стороны родственников ☐
Не было подходящей возможности ☐
Другое ☐ Укажите ______________________________

3. Какими видами секса вы **уже** занимались (выберите все подходящие ответы)

Вагинальный ☐
Анальный ☐
Оральный ☐
Предпочту не отвечать ☐

3a. Какими видами секса вы занимались в течение **прошлого года**? (Выберите все подходящие ответы)

Вагинальный ☐
Анальный ☐
Оральный ☐
Не занимался(-лась) сексом ☐
Предпочту не отвечать ☐

3b. Какими видами секса вы занимались в течение **последних четырех недель**? (Выберите все подходящие ответы)

Вагинальный ☐
Анальный ☐
Оральный ☐
Не занимался(-лась) сексом ☐
Предпочту не отвечать ☐

4. Какое из приведенных ниже утверждений лучше всего описывает человека, с которым вы последний раз занимались сексом?

   Это был человек, которого я не знал(-а) ☐
   Это был человек, с которым мы недавно познакомились ☐
   Мы были знакомы некоторое время, но не находились в постоянных отношениях ☐
   Мы раньше были в постоянных отношениях, но к этому времени уже разошлись ☐
   Мы были в постоянных отношениях на тот момент ☐
   Мы жили вместе как пара / были женаты на тот момент ☐
   Другое ☐
   Предпочту не отвечать ☐

5. В целом сколько у вас было половых партнеров, с которыми вы **когда-либо** занимались вагинальным, анальным или оральным сексом?

   1 ☐ 2 ☐ 3-4 ☐ 5-9 ☐ 10 или больше ☐

5a. Сколько половых партнеров у вас было в течение **прошлого года**?

   1 ☐ 1 ☐ 2 ☐ 3-4 ☐ 5-9 ☐ 10 или больше ☐

6. Среди ваших половых партнеров были

   Только мужчины ☐
   Только женщины ☐
   Как мужчины, так и женщины ☐ перейдите к вопросам 6a и 6b

6a. Сколько у вас было половых партнеров противоположного пола, с которыми вы когда-либо занимались вагинальным, анальным или оральным сексом? *(То есть партнеров женского пола, если вы мужчина, или партнеров мужского пола, если вы женщина)*

   1 ☐ 2 ☐ 3-4 ☐ 5-9 ☐ 10 или больше ☐

6b. Сколько половых партнеров противоположного пола у вас было в течение прошлого года?

   1 ☐ 1 ☐ 2 ☐ 3-4 ☐ 5-9 ☐ 10 или больше ☐

7. Сколько раз вы занимались вагинальным, анальным или оральным сексом в течение последних четырех недель?

   ____________________раз

(Вопрос скрыт, если респондент не занимался сексом в течение последних четырех недель)

8. Вы когда-нибудь занимались сексом за деньги, наркотики или другие ценные для вас вещи?

   Нет ☐
   Да ☐
   Предпочту не отвечать ☐

9. Вы когда-нибудь сообщали половому партнеру (партнерше) о том, что вы ВИЧ-инфицированы?

   Нет ☐
   Да ☐
   Не помню ☐

9a. Вы говорили своему нынешнему \ последнему половому партнеру о том, что вы ВИЧ-инфицированы?

Нет ☐
Да ☐
Не помню ☐

10. Вы знаете ВИЧ-статус своего нынешнего \ последнего полового партнера?

Не знаю ☐
ВИЧ-положительный ☐
ВИЧ-отрицательный ☐

11. В течение последнего года вы хоть раз занимались вагинальным или анальным сексом без презерватива?

Да — я занимался(-лась) сексом без презерватива ☐ Перейдите к вопросу 11а
Нет ☐

11а. За последний год с каким количеством человек вы занимались сексом без презерватива?

1 ☐ 2 ☐ 3-4 ☐ 5-9 ☐ 10 или больше ☐

12. Вы проверялись на наличие инфекций, передающихся половым путем, в течение последнего года?

Да ☐ Перейдите к вопросу 12а.
Нет ☐
Не знаю ☐
Предпочту не отвечать ☐

12а. Где вы проверялись?

Центр по профилактике и борьбе с ВИЧ/СПИДом ☐
В другом месте (в поликлинике, венерологической клинике, центре планирования семьи и др.) ☐

13. У вас обнаруживалась инфекция, передающаяся половым путем, в течение последнего года?

Нет ☐
Да ☐ Перейдите к вопросу 13а.
Предпочту не отвечать ☐

14. Какая инфекция (инфекции) была обнаружена? Выберите все подходящие ответы

- Хламидии ☐
- Гонорея ☐
- Сифилис ☐
- Генитальный герпес ☐
- Трихомониаз ☐
- Бактериальный вагиноз (БВ) ☐
- Генитальные (остроконечные) кондиломы ☐
- Воспалительное заболевание органов малого таза (ВЗОМТ) ☐
- Не помню названия ☐
- Другая инфекция ☐

Укажите___________________________________

15. Насколько важно для вас сейчас предохраняться от наступления беременности (у вас или у вашей партнерши)?

- Очень важно ☐
- Достаточно важно ☐
- Совсем не важно ☐

16. Вы и ваш половой партнер (партнеры), с которым (которыми) вы занимались сексом в течение последнего года, пользовались какими-нибудь методами предохранения от беременности? (Выберите все подходящие ответы)

- Презервативы ☐ Перейдите к вопросу 16а.
- Оральные контрацептивы (таблетки) ☐
- Внутриматочная спираль (ВМС) ☐
- Противозачаточные инъекции ☐
- Противозачаточные имплантаты ☐
- Противозачаточные пластыри ☐
- Прерванный половой акт ☐
- Стерилизация ☐
- Другое ☐ Укажите____________________________
- Не занимался(-лась) сексом ☐

16а. Где вы покупаете или берете презервативы? Выберите все подходящие ответы

- Аптека или обычный магазин ☐
- Торговый автомат ☐
- Бары или клубы ☐
- Центр по профилактике и борьбе с ВИЧ/СПИДом ☐
- Районная поликлиника/центр здоровья ☐
- Общественная организация ☐
- У полового партнера обычно есть презервативы ☐
- Семейные запасы ☐
- Другое ☐ Укажите___________________________

17. Вы знаете, где взять презервативы бесплатно в случае необходимости?

Да, я могу взять столько презервативов, сколько мне нужно ☐
Да, но не всегда ☐
Нет ☐

18. Вопрос только для женщин: вы когда-нибудь прерывали беременность? Если да, то сколько раз?

Никогда ☐
Да – 1 ☐
Да - 2 ☐
Да - 3 и более раз ☐

Раздел 5. Этот раздел посвящен курению, употреблению алкоголя и наркотиков

1. Как часто вы пьете напитки, содержащие алкоголь?

Никогда ☐ Перейдите к вопросу 4.
Раз в месяц или реже ☐
2-4 раза в месяц ☐
2-3 раза в неделю ☐
4 и больше раз в неделю ☐

Отвечая на следующие два вопроса, имейте в виду, что одна порция алкоголя соответствует одной бутылке пива (0,3 л), одному небольшому бокалу вина (150 мл), одной рюмке водки или одной рюмке других крепких напитков (50 мл).

2. Сколько порций алкоголя вы обычно выпиваете в день, когда вы пьете алкоголь?

1 или 2 ☐ 3 или 4 ☐ 5 или 6 ☐ 7, 8 или 9 ☐ 10 и больше ☐

3. Как часто вы выпиваете шесть или больше порций алкоголя за один день?

Никогда ☐
Реже чем один раз в месяц ☐
Каждый месяц ☐
Каждую неделю ☐
Каждый день или почти каждый день ☐

4. Вы когда-нибудь курили сигареты или табак?

Нет ☐
Да ☐ Перейдите к вопросу 4а.

4а. Вы сейчас курите сигареты или табак?

Нет ☐
Да ☐ Перейдите к вопросу 4b.

4b. Вы курите каждый день или редко?

Каждый день ☐ Укажите, сколько сигарет в день ________________________сигарет
Редко, от случая к случаю ☐

5. Вы когда-нибудь употребляли наркотики, чтобы оттянуться?

Нет ☐
Да ☐

5a. Кто-нибудь из живущих с вами употребляет уличные наркотики?

Нет ☐ Пропустите остальные вопросы этого раздела
Да ☐ Перейдите к вопросу 5b.
Предпочту не отвечать на этот вопрос ☐ Пропустите остальные вопросы этого раздела

5b. Кто из живущих с вами употребляет уличные наркотики? Выберите все подходящие ответы

Друг / сосед ☐
Партнер (или партнерша) ☐
Родитель ☐
Другой родственник ☐
Предпочту не отвечать ☐

6. В каком возрасте вы впервые попробовали наркотик?

_______________лет

7. Вы когда-нибудь считали себя зависимым от уличных наркотиков?

Нет ☐
Да ☐

8. Какие наркотики вы употребляли в прошлом? Выберите все подходящие ответы

Марихуана (каннабис, гашиш) ☐
Героин ☐
Опиаты, изготовленные в домашних условиях («ширка», «ханка») ☐
Амфетамины («винт», «джефф» и т.д.) ☐
Экстази ☐
Дезоморфин («крокодил») ☐
Мефедрон («соль для ванны») ☐
Уличный метадон ☐
Бензодиазепины ☐
Другое ☐ Укажите_________________________________

9. Вы когда-нибудь (или в настоящее время) кололи себе наркотики?

Да, в прошлом ☐ Перейдите к вопросам 9a, 9b
Да, сейчас ☐ Перейдите к вопросам 9a, 9b
Нет, я никогда не колол(-а) себе наркотики ☐

9a. В каком возрасте вы впервые укололи себе наркотик?

_______________лет

9b. Кто сделал вам первый укол наркотика?

- Продавец (дилер) ☐
- Брат или сестра ☐
- Друг ☐
- Партнер ☐
- Я сам(-а) ☐
- Кто-то другой ☐ Укажите ________________

11. Вы сейчас употребляете уличные наркотики?

- Нет ☐
- Да ☐ Перейдите к вопросу 11а.
- Предпочту не отвечать ☐

11а. Какие наркотики вы сейчас употребляете?

- Марихуана (каннабис, гашиш) ☐
- Героин ☐
- Опиаты, изготовленные в домашних условиях («ширка», «ханка») ☐
- Амфетамины («винт», «джефф» и т.д.) ☐
- Экстази ☐
- Дезоморфин («крокодил») ☐
- Мефедрон («соль для ванны») ☐
- Уличный метадон ☐
- Бензодиазепины ☐
- Другое ☐ Укажите________________________________

12. Вы когда-нибудь стояли на учете в наркодиспансере?

- Нет ☐
- Да ☐

13. Вы когда-нибудь участвовали в программах снижения вреда для потребителей наркотиков?

- Нет ☐
- Да ☐ Перейдите к вопросу 14а.

13а. Какую помощь вы получали в рамках программ снижения вреда?

- Стерильные иглы/шприцы ☐
- Презервативы ☐
- Консультации по предохранению от беременности ☐
- Тесты на беременность ☐
- Тестирование на ВИЧ ☐
- Тестирование на другие инфекции, передающиеся половым путем ☐
- Информация и направление в службы лечения ВИЧ-инфекции ☐
- Информация и направление в программы заместительной терапии метадоном/бупренорфином ☐
- Вакцинация против гепатита В ☐
- Другое ☐ Укажите ________________

14. Вы когда-нибудь лечились от наркотической зависимости? Выберите все подходящие ответы

Абстиненция (детоксикация/реабилитация) ☐

Заместительная терапия метадоном/бупренорфином ☐ Перейдите к вопросам 14a, 14b, 14c, 14d

Ничего из этого ☐

14a. Вы сейчас получаете метадон или бупренорфин в рамках лечения от наркотической зависимости?

Нет ☐

Да ☐

14b. Как часто вы принимаете метадон/бупренорфин (или принимали в прошлом)?

Ежедневно ☐

Каждые 2 дня ☐

Другое ☐ Укажите _________________

14c. Где вы получаете метадон/бупренорфин (или получали в прошлом)?

Наркологическая клиника ☐

Центр по профилактике и борьбе со ВИЧ/СПИДом ☐

Пункт продажи бупренорфина ☐

Другое ☐ Укажите _________________

14d. Сколько времени у вас обычно занимает дорога до места выдачи заместительного препарата (метадона или бупренорфина)?

_______часов_______ минут

15. Были ли какие -то вопросы для Вас непонятными или сложными?

Нет ☐

Да ,один два вопроса ☐ Перейдите к вопросам 15a

Да, более два вопросов ☐ Перейдите к вопросам 15a

15a. обращались ли Вы к кому-нибудь за помощью и разъяснениями?

Нет ☐

Да ☐

Огромное спасибо за заполнение этого опросника!

Если у вас есть какие-либо вопросы или сомнения по поводу любой темы, затронутой в этом опроснике, или если вы хотите что-то обсудить, пожалуйста, обратитесь к своему врачу. На выданном вам информационном листке есть список организаций, предоставляющих информацию и поддержку молодым людям, живущим с ВИЧ-инфекцией.

Нам бы хотелось рассказать ВИЧ-инфицированным молодым людям о результатах этого исследования. Как вы думаете, как лучше это сделать?

Повесить плакат и раздавать информационные листки и в Центре профилактики и борьбы с ВИЧ/СПИДом ☐

Разместить информацию на сайте в интернете ☐
Пригласить молодых людей на встречу для обсуждения результатов исследования ☐
Другое – пожалуйста, напишите свое предложение____________________________________
